# Continuous-wave perovskite polariton lasers

**DOI:** 10.1126/sciadv.adr8826

**Published:** 2025-01-10

**Authors:** Chen Zou, Xuhui Cao, Zixiang Wang, Yichen Yang, Yaxiao Lian, Baodan Zhao, Dawei Di

**Affiliations:** State Key Laboratory of Extreme Photonics and Instrumentation, College of Optical Science and Engineering, International Research Center for Advanced Photonics, Zhejiang University, Hangzhou 310027, China.

## Abstract

Solution-processed semiconductor lasers are next-generation light sources for large-scale, bio-compatible and integrated photonics. However, overcoming their performance-cost trade-off to rival III-V laser functionalities is a long-standing challenge. Here, we demonstrate room-temperature continuous-wave perovskite polariton lasers exhibiting remarkably low thresholds of ~0.4 W cm^−2^, enabled by a variable single-crystal perovskite microcavity. The threshold outperforms state-of-the-art III-V lasers by ~30 times under optical pumping, and is exceptional among solution-processed lasers. The ultralow-threshold lasing arises from steady-state exciton-polariton condensation, a macroscopic quantum phenomenon akin to Bose-Einstein condensation. The steady-state condensation is attained by fine-tuning the cavity photon-exciton energy separation near the degeneracy point for strong light-matter interactions. These mechanisms enabled the initial demonstration of an indirectly injected perovskite laser chip powered by a gallium nitride light-emitting diode. Our findings create exciting avenues toward on-chip integration of solution-processed lasers, opening opportunities for lasing with ultralow energy consumption and unprecedented performance.

## Teaser

Room-temperature steady-state polariton condensation enables CW perovskite lasers with ultralow thresholds.

## INTRODUCTION

Solution-processed semiconductor lasers, including those based on organic (*1*–*5*), colloidal quantum-dot (*6*–*9*), and halide perovskite (*10*–*14*) materials, show strong promises as a generation of coherent light sources for large-area, flexible and integrated photonics. Among the lasing materials, halide perovskites have been identified as an exceptional class (*10*–*17*); they exhibit a range of desirable properties including strong optical gain, high emission quantum yields, tunable wavelengths, and the ease of preparation, as were similarly found in other optoelectronic applications (*18*–*23*). In recent years, room-temperature continuous-wave (CW) lasing from halide perovskites was demonstrated (*12*, *24*, *25*). These lasers generally showed low thresholds of 13 to 45 W cm^−2^, with a few exceptions (*26*) reporting lower thresholds but unconfirmed coherence properties.

Despite the encouraging advances, the performance of perovskite lasers (or solution-processed lasers in general) is typically inferior to that of the best III-V semiconductor lasers, which show thresholds of down to ~12 W cm^−2^ under optical pumping (*27*). Further development in this direction is limited by the trade-off between quality and processing simplicity of solution-processed semiconductors.

**Fig. 1.**
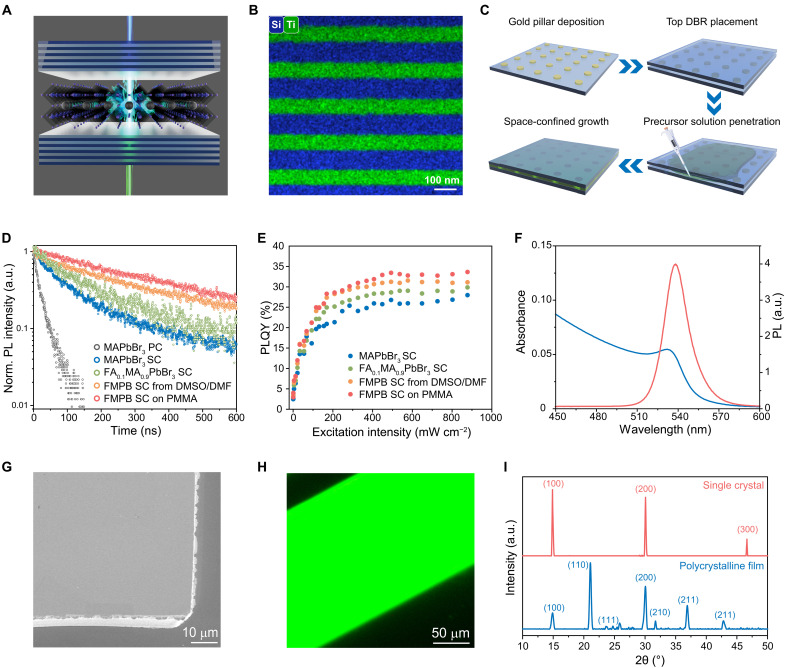
Fabrication and material characterization of the perovskite polariton lasers. (**A**) Schematic representation of a CW perovskite polariton laser. A perovskite single crystal is sandwiched between a pair of highly reflective DBRs, supporting CW lasing action through strong light-matter interactions. (**B**) Scanning transmission electron microscopy (STEM) and energy-dispersive spectroscopy (EDS) images of the DBR, showing bilayers of SiO_2_ (blue regions) and TiO_2_ (green regions). (**C**) Fabrication processes of the single-crystal perovskite microcavities. (**D**) Transient PL decay curves of perovskite samples (excitation wavelength: 400 nm; fluence: 42 nJ cm^−2^). FMPB denotes FA_0.1_MA_0.9_PbBr_3_ perovskite. SC denotes single-crystal samples. PC denotes polycrystalline samples. (**E**) PLQY as a function of excitation intensity for various perovskite samples. (**F**) Absorption and PL spectra of a FA_0.1_MA_0.9_PbBr_3_ single crystal (thickness: ~200 nm). (**G**) Scanning electron microscopy (SEM) image of a FA_0.1_MA_0.9_PbBr_3_ single crystal. (**H**) Fluorescent microscope image of a FA_0.1_MA_0.9_PbBr_3_ perovskite single crystal. (**I**) XRD patterns of FA_0.1_MA_0.9_PbBr_3_ single crystal and polycrystalline samples.

Here, we demonstrate CW perovskite polariton lasers, achieving a remarkably low lasing threshold of ~0.4 W cm^−2^ at room temperature. The laser construction consists of a perovskite single crystal contained within a variable Fabry-Perot micro-resonator (Fig. 1A). The lasing thresholds are more than one order of magnitude lower than those achieved in state-of-the-art III-V semiconductor lasers under optical pumping (*27*–*29*), and are exceptional among solution-processed lasers.

Beyond our experiments that rigorously demonstrate the key attributes of lasing (*30*), we find that the ultralow threshold originates from exciton-polariton condensation (*17*, *31*–*38*), a macroscopic coherent quantum state resembling a Bose-Einstein condensate (BEC) (*31*, *33*, *39*) operating in the steady state; this is a condition not achieved before for solution-processed semiconductors. The BEC-like state is obtained through an optimum cavity photon-exciton energy separation near the degeneracy point, allowing the strong coupling of light and matter in the perovskite single-crystal microcavity. The polariton condensation is found to markedly reduce the lasing threshold by two orders of magnitude compared to that of conventional photon lasing for the same gain medium. The extremely low threshold leads to the realization of an indirectly injected perovskite laser chip (PeLC) driven by a gallium nitride (GaN) light-emitting diode (LED).

## RESULTS

### Fabrication and material characterization of the CW perovskite polariton lasers

The microcavity used in this work consists of a mixed-cation lead bromide perovskite single crystal sandwiched between two distributed Bragg reflectors (DBRs) with alternating layers of SiO_2_/TiO_2_ (Fig. 1, A and B). The perovskite single crystals were prepared using a space-confined inverse-temperature crystallization method (*40*–*43*) (Fig. 1C and Materials and Methods). An array of gold pillars (diameter: 1 mm, period: 3 mm) were thermally evaporated onto a DBR, followed by the placement of another DBR. The gold pillars act as a spacer between the two DBRs, forming empty channels for single crystal growth. The precursor solution was introduced into the channels, followed by annealing at 80°C to form the single crystals. The thickness of the perovskite single crystals (and the cavity lengths) could be freely tuned between 0.1 and 1.5 μm (fig. S1) by adjusting the height of the evaporated gold pillars.

Halide perovskites with mixed A-site cations were reported to demonstrate superior optoelectronic properties and stability (*44*). We find that, by introducing formamidinium (FA) into the perovskite composition (to form FA*_x_*MA_1−*x*_PbBr_3_), using mixed solvents of dimethyl sulfoxide (DMSO) and dimethylformamide (DMF), and introducing a hydrophobic polymethyl methacrylate (PMMA) layer (between the perovskite and the bottom DBR), it is possible to obtain perovskite single crystals with further refined quality (fig. S2). This method was improved upon earlier works using hydrophobic substrates to reduce the density of nucleation sites and alter the crystallization kinetics for improved single crystal growth (*40*). From our nuclear magnetic resonance (NMR) results, the molar ratios of FA^+^ to MA^+^ in the precursor solution are found to closely agree with the actual stoichiometric ratios of the corresponding cations in the perovskite single crystals (fig. S3). The optimized FA_0.1_MA_0.9_PbBr_3_ single crystals show a low trap density of ~8.7 × 10^13^ cm^−3^ (four orders of magnitude lower than that of polycrystalline FA_0.1_MA_0.9_PbBr_3_ films, fig. S4), a long PL lifetime of >400 ns (Fig. 1D), and a high external photoluminescence quantum yield (PLQY) of ~33.4% (Fig. 1E).

The optical absorption and PL of the FA_0.1_MA_0.9_PbBr_3_ single crystals show small Stokes shift and substantial spectral overlap (Fig. 1F). The absorption curves can be modeled using the Elliott formula (see note S1 and fig. S5) (*45*), revealing an exciton binding energy of ~41 meV and an exciton peak position of ~2.32 eV. The exciton binding energy is slightly larger than the thermal energy at room temperature (26 meV). In this case, correlated and uncorrelated e-h pairs (i.e., excitons and free carriers) are expected to coexist in thermal equilibrium under excitation intensities near which polariton lasing can occur (10^15^ to 10^18^ cm^−3^), according to the Saha equation (see note S2 and fig. S6) (*15*). The FA_0.1_MA_0.9_PbBr_3_ single crystals exhibit uniform surface morphology free from macroscopic dislocations (Fig. 1G), showing an RMS roughness of ~0.6 nm (fig. S2), allowing minimum optical scattering losses as an active medium for polariton lasing. In contrast, a higher surface roughness (RMS) of ~6.8 nm is observed for polycrystalline samples. The FA_0.1_MA_0.9_PbBr_3_ single crystals show bright and uniform photoluminescence (PL) under UV illumination (Fig. 1H). The perovskite single crystals exhibit a cubic phase (space group *Pm*3*m*) with <100> orientation (Fig. 1I). They show sharper and fewer x-ray diffraction (XRD) peaks compared to those of polycrystalline films with a similar composition, consistent with the higher crystallinity of the single crystals.

### Demonstration of CW lasing from polariton condensation

**Fig. 2.**
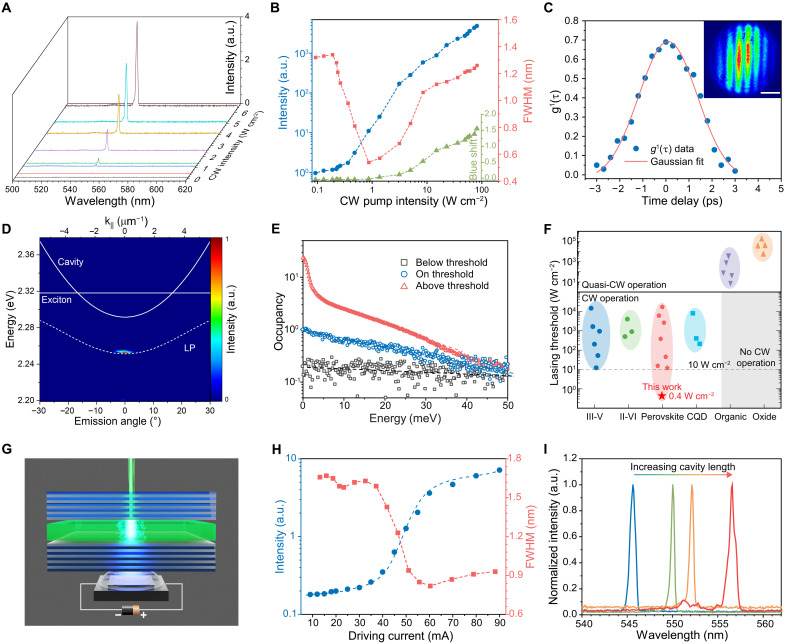
CW polariton lasing from single-crystal perovskite microcavities at room temperature. (**A**) Evolution of emission spectra under CW operation at different pump intensities. Single-crystal sample thickness: ~120 nm. (**B**) Light-output intensity, FWHM, and emission blue shift as functions of CW pump intensity (at ~300 K). (**C**) Temporal coherence of the CW polariton laser. The first-order coherence *g*^1^(τ) above the threshold, measured using a Michelson interferometer. The red solid line represents a Gaussian fit to the data. The inset shows the Michelson interference pattern at zero time delay. Scale bar, 5 μm. (**D**) The angle-resolved PL spectra above the lasing threshold (3.0*P*_th_). The straight line represents the dispersion relation of excitons. The solid parabolic curve represents the dispersion relation of cavity photons. The dashed curve represents the dispersion of the lower polariton branch. (**E**) Polariton occupancy in the ground and excited states, plotted in a semilogarithmic scale for pump intensities below (0.8*P*_th_), around (~1.0*P*_th_), and above (3.0*P*_th_) the threshold (at ~300 K). The blue dashed line represents the Maxwell–Boltzmann distribution function with *k*_B_*T* = 26 meV. (**F**) Comparison of CW lasing thresholds for lasers based on different classes of semiconductor materials. See table S1 for details. (**G**) Schematic illustration of an indirectly injected perovskite laser chip, powered by a 410-nm GaN LED. (**H**) Emission intensity and FWHM of the perovskite polariton laser chip as functions of driving current in the GaN LED (dc, at ~300 K). (**I**) Polariton lasing spectra from microcavities of variable lengths (adjusted using the single-crystal thickness). The second peak/shoulder in the spectrum of the device with the longest cavity length (red curve) can be attributed to the leakage of cavity-mode photons. The spectral linewidths are related to the detuning of the cavity.

To characterize the lasing properties, the single-crystal perovskite microcavities were pumped off resonance using a 405-nm CW diode laser. As the excitation intensity increases to above ~0.4 W cm^−2^, the emission peak at ~551 nm rises sharply (Fig. 2A). The real-space images of the light output (fig. S7) show a transition from weak diffuse luminescence to high-brightness concentrated emission. The emission intensity as a function of excitation intensity is plotted on a log-log scale (Fig. 2B), showing a threshold (*P*_th_) of 0.4 W cm^−2^. The emission intensity from the microcavity increases sub-linearly below the threshold; this may be attributed to self-reabsorption losses inside the cavity (*46*). The increase becomes super-linear and quadratic above the threshold. In the regime closer to saturation, the increase of emission intensity becomes linear. Narrowing of linewidth from ∼1.3 to ∼0.54 nm across the threshold is observed (Fig. 2B), accompanied by a blue shift (~1.5 nm) of the emission peak. The emission spectra gradually broaden under higher pump intensities, likely owing to the fluctuation of polariton population and saturation of occupied ground states. The temporal coherence properties of the emission from the microcavities were examined using a Michelson interferometer. The first-order coherence function *g*^1^(τ) (Fig. 2C) is given by the visibility of the interference fringes. At zero time-delay (τ = 0 ps) above the threshold, the collected interference pattern shows a maximum fringe visibility contrast of 69% (Fig. 2C, inset). The clear threshold (Fig. 2B), linewidth narrowing (Fig. 2B), beam profiles (fig. S7), polarization of emission (fig. S8), and temporal coherence (Fig. 2C and fig. S9) are consistent with the characters of lasing according to well-established measurement protocols (*30*). The perovskite lasers show decent operational lifetimes (*T*_50_) of ~6.4 hours under continuous pumping with an intensity of 0.9 W cm^−2^ (fig. S10).

The occurrence of steady-state polariton condensation responsible for the ultralow-threshold lasing can be observed through angle-resolved emission measurements at room temperature (see Fig. 2D for above-threshold condition and fig. S11 for below- and near-threshold conditions). The solid curves in Fig. 2D are the dispersion relations of the uncoupled exciton (*E*_ex_ = 2.32 eV) and cavity photon modes $\left[E_{\text{cav}}\left(\theta \right)=E_{\text{cav}}\left(0\right){\left(1-{\text{sin}}^{2}\theta /n_{\text{eff}}^{2}\right)}^{-1/2}\right]$, where θ is the emission angle and *n*_eff_ is the effective refractive index]. The dashed curve shows the dispersion relation of the lower polariton (LP) branch from the coupled harmonic oscillator mode (*31*, *47*), which can be described by $E_{\text{LP}}=\frac{\left(E_{\text{cav}}+E_{\text{ex}}\right)-\sqrt{{\left(E_{\text{cav}}-E_{\text{ex}}\right)}^{2}+{\left(\hbar \Omega \right)}^{2}}}{2}$, where ℏΩ is the Rabi splitting energy that is found to be ~115 meV (see note S3 and fig. S12). The ground state near the minimum of the LP dispersion [${E_{\text{LP}}}_{\left(k_{\|}=0\right)}$ = 2.251 eV, where *k*_||_ is the in-plane wave vector] becomes massively occupied, in clear contrast to conventional photon lasing in which an occupation of the minimum of the cavity-mode dispersion curve [${E_{\text{cav}}}_{\left(k_{\|}=0\right)}$= 2.292 eV] is expected. The enhanced ground-state occupation at the bottom of the polariton dispersion relation is a defining feature of polariton condensation (*14*, *34*). In contrast, for a weakly coupled perovskite microcavity with poorer optical quality (fig. S13), only cavity modes are seen in the angle-resolved emission spectra (fig. S14) without any evidence for lasing.

The occupancy of polaritons as a function of their energy (relative to the ground state) at different pump intensities is shown in Fig. 2E. At very low excitation intensities, the polariton occupancy is not thermalized (*31*). At the lasing threshold, the occupancy function can be modeled using a Maxwell–Boltzmann distribution: $n\left(E\right)/\text{exp}(-∆E/k_{B}T)$, where $∆E=E_{k_{\|}}-E_{k_{\|}=0}$ and *k*_B_ is Boltzmann’s constant, indicating thermal equilibrium of polaritons at room temperature (*31*, *33*). Above the threshold, a peak in the energetic distribution near *k*_||_ = 0 can be observed, consistent with the formation of polariton condensation near the ground state of the LP dispersion (*14*, *34*).

Together, the blue shift of emission wavelengths (Fig. 2B), narrowing of the emission spectra in the energy and momentum spaces (Fig. 2B and fig. S11), and the excitation-dependent occupancy functions (Fig. 2E) indicate that the low-threshold CW lasing arises from steady-state polariton condensation, a coherent quantum state similar to the BEC (*33*, *36*).

### Ultralow threshold and an indirectly injected PeLC

The thresholds of ~0.4 W cm^−2^ for the CW perovskite polariton lasers are significantly lower than those of the state-of-the-art inorganic semiconductor lasers based on III-V, II-VI, and oxide materials under optical pumping, and are exceptional for solution-processed lasers based on organic, colloidal quantum dot (CQD), and perovskite semiconductors (Fig. 2F and table S1). The perovskite polariton lasers are highly reproducible, showing an average CW lasing threshold of 0.76 W cm^−2^ and an SD of 0.19 W cm^−2^ (fig. S15).

The ultralow-threshold polariton lasing allowed us to demonstrate an indirectly injected PeLC. The PeLC was constructed using a single-crystal perovskite microcavity directly attached to a GaN LED (peak wavelength: 410 nm; maximum power: 3 W; driven by dc source) (Fig. 2G and fig. S16). A drastic increase of the output intensity from the microcavity was observed for driving currents of above ~41 mA, accompanied by a clear linewidth narrowing from ~1.6 to ~0.8 nm across the threshold (Fig. 2H). The evolution of emission spectra at increasing driving current, polarization characteristics above the threshold, and angle-resolved emission spectra support the occurrence of polariton lasing from the PeLC (fig. S16). The lengths of the microcavities could be tuned by varying the perovskite single crystal thickness. Single-mode lasing with variable wavelengths from 545 to 557 nm was achieved (Fig. 2I). The fact that CW lasing can be achieved with an indirectly injected configuration (*48*) under low driving currents of ~41 mA (~4.1 A cm^−2^) indicate the exceptional potential of perovskite polariton lasers as on-chip photon sources.

### The transition from polariton to photon lasing

**Fig. 3.**
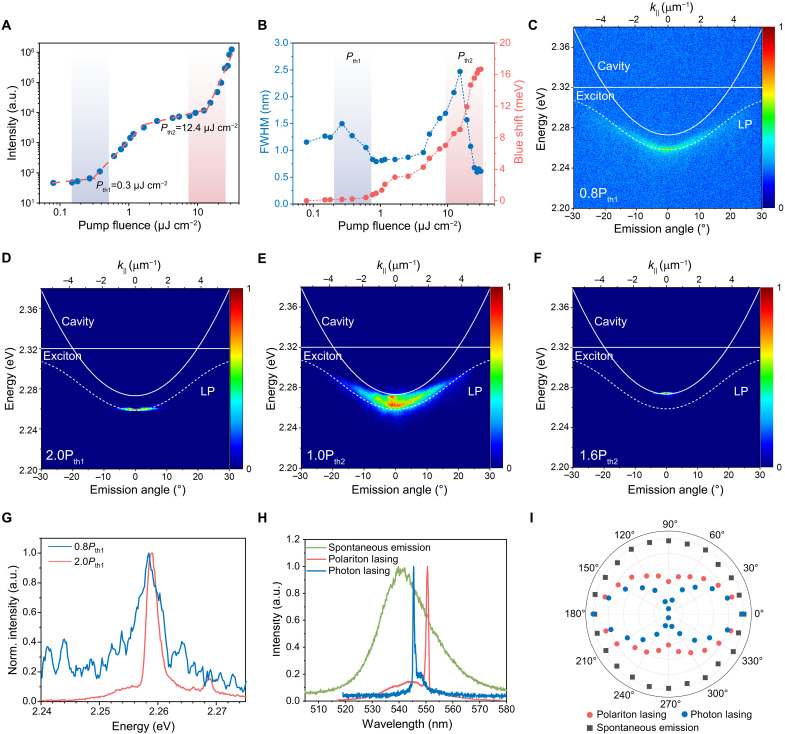
Polariton to photon lasing transition in single-crystal perovskite microcavities. (**A**) Emission intensity (*I*)–pump fluence (*I*_0_) curve of a single-crystal perovskite microcavity, showing two thresholds at pump fluence of 0.3 μJ cm^−2^ and 12.4 μJ cm^−2^ corresponding to polariton lasing and photon lasing, respectively. The samples were pumped using a 400-nm fs laser (pulse width: 270 fs, repetition rate: 50 kHz, single-crystal thickness: ~130 nm). Above the first threshold, the increase in output intensity is super-linear with a power-law dependence of $I\propto {I_{0}}^{2.36}$. In the regime where photon lasing (light amplification by stimulated emission) occurs, the intensity increases more sharply with $I\propto {I_{0}}^{6.67}$. (**B**) FWHM and wavelength blue shift as functions of pump fluence, showing marked changes at the two thresholds. The shaded areas are regions near the thresholds. (**C** to **F**) Angle-resolved emission spectra of a single-crystal perovskite microcavity at different pump fluences: (C) *P* = 0.8*P*_th1_, (D) *P* = 2*P*_th1_, (E) *P* ~ *P*_th2_, and (F) *P* = 1.6*P*_th2_. (**G**) Emission spectra under pulsed pumping at 0.8*P*_th1_ and 2.0*P*_th1_. The emission blue shift (by ~0.6 meV) and linewidth narrowing (by ~2.3 meV) can be observed. (**H**) Spectra of spontaneous emission, polariton lasing, and photon lasing from the single-crystal perovskite. (**I**) The polarization characteristics.

Beyond the aforementioned steady-state (CW pumped) experiments, we extend our investigations into the pulsed mode (using a femtosecond laser) where excitation intensities exceeding the damage threshold of CW operation (~400 W cm^−2^) can be accessed. This allows us to observe the transition from polariton to photon lasing using a series of pump fluences (Fig. 3A). Under pulsed excitation, the threshold of polariton lasing (*P*_th1_) is about 0.3 μJ cm^−2^, which is about two orders of magnitude smaller than the threshold of photon lasing (*P*_th2_ = 12.4 μJ cm^−2^). Similar to what was observed for CW operation, the nonlinear rise of output intensity is accompanied by a blue shift of peak wavelengths and linewidth narrowing (Fig. 3B). The linewidth of the polariton emission reduces across the threshold; it broadens again as the pump fluence further increases. A blue shift of emission (~1.3 nm) relative to the minimum of the LP branch, a signature of polariton lasing, is observed. These phenomena could be attributed to polariton-polariton interactions (*49*). As the pump fluence further increases, the emission energy exhibits another substantial blue shift toward the minimum of the cavity-mode dispersion relation (2.273 eV). The second region of linewidth narrowing near *P*_th2_ = 12.4 μJ cm^−2^ is also observed.

Angle-resolved emission spectra of the single-crystal perovskite microcavities at different regimes of operation are presented in Fig. 3 (C to F). Below the polariton lasing threshold (*P*_th1_), the polariton emission follows the profile of the LP dispersion over a wide range of angles (Fig. 3C). Under excitation intensities above *P*_th1_, the polaritons condense into the ground state, a much narrower energetic and angular distribution at the bottom of LP branch is observed (Fig. 3D). Correspondingly, clear blue shift and linewidth narrowing of the emission peak are observed (Fig. 3G), consistent with the occurrence of polariton condensation. As the exciton density increases toward the Mott density, the Coulomb interactions required for stable excitons are weakened, creating an electron-hole plasma (*45*, *50*). When the excitation fluence increases toward the second threshold (*P*_th2_), a second emission peak corresponding to the cavity photon mode appears, indicating the simultaneous presence of cavity photon and polariton modes as the system transits from strong coupling to a weak coupling regime (Fig. 3E). Photon lasing occurs at above the second threshold (*P*_th2_), with the energy and momentum of emission restricted to near the bottom of the cavity-mode dispersion relation (Fig. 3F).

The spontaneous emission, polariton lasing, and photon lasing from the perovskite single crystals show distinctively different spectra (Fig. 3H). The spontaneous emission from a bare perovskite single crystal is centered at ∼2.305 eV with a full width at half maximum (FWHM) of ~22 nm. When assembled into a microcavity, under low to medium excitation intensities (0.1 to 10 μJ cm^−2^), the emission of ground-state polariton is located at ~2.258 eV with a narrowing of linewidth to ~0.8 nm. When the pump fluence exceeds the threshold of photon lasing, the emission FWHM continues to reduce to ~0.5 nm. The emission peak blue shifts to 2.273 eV at the same time.

Further, we compare the polarization properties of the spontaneous emission, polariton lasing, and photon lasing (Fig. 3I). Below the polariton lasing threshold (*P*_th1_), the spontaneous emission is completely unpolarised. Above *P*_th1_, the high density of polaritons arising from the strong coupling of cavity photons and excitons create a BEC-like polariton condensation with macroscopic coherence (*34*). The light emitting from the microcavity shows a degree of linear polarization of (*I*_max_ − *I*_min_)/(*I*_max_ + *I*_min_) ≈ 42.9% (*I*_max_ is the maximum intensity at 0°, and *I*_min_ is the minimum intensity at 90°). As the pump fluence increases to above the photon lasing threshold (*P*_th2_), a higher degree of linear polarization (~81.8%) is achieved. We note that the polariton lasing shows a smaller degree of polarization compared to that of photon lasing, consistent with that reported for polariton lasing from III-V and organic semiconductors (*51*, *52*).

### The influence of detuning energy

We show below that detuning, the cavity photon-exciton energy separation ($∆=E_{\text{cav}}-E_{\text{ex}}$), is a key factor in enabling the ultralow threshold of polariton lasing from single-crystal perovskite microcavities. Using our method of perovskite single crystal growth (Materials and Methods), the cavity length and the cavity mode position can be precisely controlled by the thickness of the single crystals, leading to finely adjustable detuning energies.

**Fig. 4.**
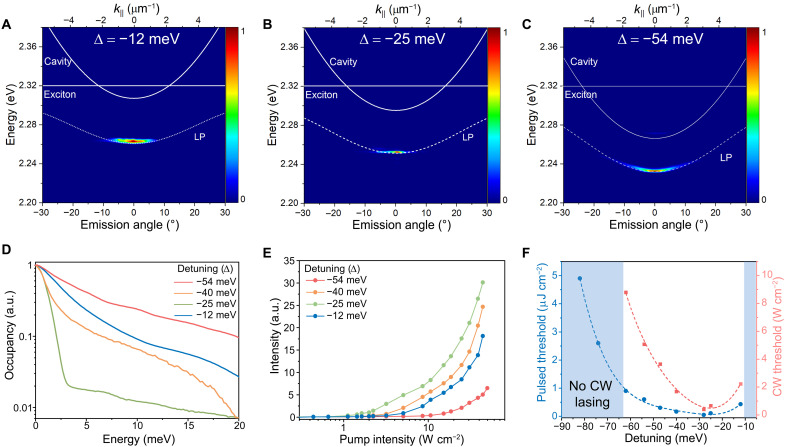
The effects of detuning energy on polariton lasing. (**A** to **C**) Angle-resolved emission spectra of single-crystal perovskite microcavities for different detuning energies of (A) ∆ = −12, (B) ∆ = −25, and (C) ∆ = −54 meV (at *P* = 3*P*_th_). The thicknesses of single-crystal samples in (a)-(c) are 100 nm, 120 nm and 140 nm, respectively. (**D**) Polariton occupancy functions for different detuning energies (at *P* = 3*P*_th_). (**E**) The emission intensity-pump intensity characteristics for different detuning energies (∆ = −54, −40, −25, and −12 meV). (**F**) Dependence of lasing threshold on detuning energy under pulsed (blue curve) and CW (red curve) pumping. CW polariton lasing is not observed for ∆ < −62 meV and ∆ > −10 meV, as shown in blue grade regions.

The angle-resolved emission spectra from the single-crystal perovskite microcavities with different detuning energies are shown in Fig. 4 (A to C). At ∆ = −54 meV, the angular distribution of emission is relatively broad along the LP branch, indicating a considerable fraction of uncondensed polaritons. At ∆ = −25 and −12 meV, clear reductions in the energy and angular distributions toward the LP ground state are observed, consistent with the formation of polariton condensation. A similar finding could be observed in the energy-dependent occupancy of polaritons at different detuning energies (Fig. 4D). In the case of large detuning (∆ = −54 meV), a polariton relaxation bottleneck is observed at excited states and polaritons tend to be resident at a broad range of energies (*52*). This may be related to inefficient polariton-polariton scattering processes for polaritons with a larger photon fraction, hindering polariton condensation (*53*). At smaller negative detuning (∆ = −40, −25, and −12 meV), the increased excitonic component in polaritons leads to the more efficient relaxation of polaritons.

The output intensity-pump fluence characteristics of the single-crystal perovskite microcavities are compared for different detuning energies (Fig. 4E). As the excitonic fraction increases (with less negative ∆), the lasing threshold decreases from ∼8.8 W cm^−2^ at a detuning of −62 meV to 0.4 W cm^−2^ at a detuning of −28 meV under CW operation (a similar trend is observed under pulsed pumping) (Fig. 4F). However, further minimizing the detuning energy leads to slightly increased thresholds, possibly due to the unfavorable curvature at the bottom (*k* ~ 0) of the LP dispersion for very small detuning (*52*). CW polariton lasing is not observed for ∆ < −62 meV and ∆ > −10 meV, where the strong coupling between excitons and photons cannot be sustained. The analysis above exemplifies how detuning energies are optimized in the single-crystal perovskite microcavities for ultralow-threshold polariton lasing.

## DISCUSSION

In summary, we have demonstrated CW perovskite polariton lasers, constructed using high-quality mixed-cation perovskite single crystals placed within a finely adjustable Fabry-Perot microcavity. They show exceptionally low CW lasing thresholds of ~0.4 W cm^−2^ at room temperature, which are an order of magnitude (~30 times) lower than those achieved in state-of-the-art III-V semiconductor lasers under optical pumping, and are exceptional among solution-processed lasers (Fig. 2F and table S1). The clear threshold (Fig. 2B), linewidth narrowing (Fig. 2B), beam profiles (fig. S7), the polarization of emission (fig. S8), and the temporal coherence (Fig 2C and fig. S9) clearly demonstrate the occurrence of lasing (*30*).

Additional characteristics of the perovskite microcavity, including blue shift of the emission wavelengths (Fig. 2B), narrowing of the emission spectra in the energy and momentum spaces (Fig. 2D and fig. S11), the excitation-dependent occupancy functions (Fig. 2E), and the sub-Mott density carrier concentration (fig. S6) show that the ultralow threshold arises from exciton-polariton condensation (note S4), a macroscopic coherent quantum state akin to a BEC (*33*, *36*) operating in the steady state; this is a regime not formerly accessible for solution-processed semiconductors. Such an ideal condition is established by adjusting the cavity photon-exciton energy separation near the degeneracy point, allowing strong light-matter interactions in the single-crystal microcavity. The polariton condensation reduces the lasing threshold by two orders of magnitude compared to that of conventional photon lasing for the same perovskite single crystals. These mechanisms lead to the initial demonstration of an indirectly injected PeLC powered by a GaN LED. We expect our findings to create exciting opportunities for the on-chip integration of solution-processed lasers for emerging applications, and a room-temperature, quasi-equilibrium platform for polariton BECs.

## MATERIALS AND METHODS

### Fabrication of CW perovskite polariton lasers

Alternating layers of silicon dioxide (SiO_2_, *n* = 1.478) and titanium nitride (TiO_2_, *n* = 2.498) were sputtered onto the glass substrate to form DBRs. Mixed MABr, FABr, and PbBr_2_ in different molar ratios were fully dissolved in a mixed solvent of DMF/DMSO (volume ratio = 4:1) to obtain a 1.2 M perovskite precursor solution. A thin PMMA layer (10 nm) was spin coated onto each DBR substrate to improve the surface hydrophobicity. Au pillars (period: 3 mm; diameter: 1 mm) were evaporated onto the bottom DBR substrate as a spacer with a variable height of 0.1 to 1.5 μm. A pair of DBRs were pressed and bonded together by a custom-made clip to form empty channels. A drop of perovskite precursor solution (5 μl) was then injected from the edge of the DBR pair, penetrating into the channels due to the capillary force. The single crystal growth experiments were done in air. The annealing temperature for the samples was gradually increased from room temperature to 80°C at a rate of 2°C/min and then kept at 80°C for 24 hours. The total duration of the crystal growth was about 2 days. To directly measure the properties of the single crystals, the top DBR was removed by inserting a thin razor blade between the DBR and the top surface of the perovskite crystal. The lateral length of each perovskite single crystal exhibiting lasing is normally less than 0.4 mm. The average separation between a gold pillar and a perovskite single crystal is orders of magnitude longer than the emission wavelength (~550 nm) of the perovskite, suggesting that plasmonic modes are unlikely a key contributor to the lasing performance.

### Surface morphology and thickness analyses

The surface morphology of the perovskite single crystals grown on DBR substrates was inspected using a field emission scanning electron microscope (Hitichi, SU70 SEM). The surface roughness of the samples was characterized using an atomic force microscope (Bruker, Multimode-8) under the tapping mode. The thickness of perovskite single crystals was measured using a stylus profilometer (Bruker, DektakXT).

### STEM and EDS measurements

A spherical aberration-corrected scanning transmission electron microscopy (FEI, Titan ChemiSTEM) was used for collecting the cross-sectional images of the devices and the EDS mapping data of the DBRs. The samples for HAADF-STEM measurements were prepared using a dual-beam focused-ion-beam system (Quata 3D FEG).

### Absorption and PL measurements

The ultraviolet–visible absorption spectra of the perovskite single crystals were measured using a double beam spectrophotometer (Aoyan UV1901PC) in ambient conditions. The spot size of the probe light in the spectrometer was 9 mm^2^, which was larger than the lateral area of each crystal (~0.3 mm^2^). A 405-nm CW laser was used to excite the perovskite single crystals, and the PL spectra were obtained by a high-resolution spectrometer (QE-Pro, Ocean Optics).

### PLQY measurements

The PLQYs of perovskite single crystal and polycrystalline films were measured using an integrating sphere. A 405-nm CW laser whose emission power can be adjusted was used as the excitation source. The PLQYs were calculated from the ratio of the number of photons from the PL to the number of absorbed photons from the pump laser.

### XRD measurements

The XRD patterns of the samples were collected by an x-ray diffractometer (Shimadzu XRD 7000) using Cu Kα_1,2_ radiation (λ = 1.541 Å). The measurements were performed under the continuous mode with a scan range of 5° < 2θ < 50° and a scan speed of 5° per minute.

### Time-resolved PL measurements

The PL from the samples was directed into a fiber-coupled avalanche photodiode (APD; ID100, IDQ). The time-resolved PL measurements were performed using a time-correlated single-photon counting (TCSPC) system (PicoHarp 300 counter, PicoQuant) while the perovskite samples were photo-excited by a Yb^3+^: YAG femtosecond laser (400 nm, ~270 fs, 50 kHz; Pharos, Light Conversion Ltd.). For experiment at low temperature, the sample was placed in a cryostat with temperature controlled from 80 to 300 K.

### Angle-resolved emission experiments

The angle-resolved measurements were performed using an inverted microscope (Olympus). The angle resolved emission mapping was measured in a custom-built micro-PL setup with a Fourier imaging configuration. A high–numerical aperture (NA) 50× microscope objective (NA = 0.6) was used in our angle-resolved measurement, covering an angular range of ±36°. Emission from the samples was collected through the same objective and directed through a 450-nm long-pass dichroic beam splitter. Two 75-mm Fourier lenses were used to project the back aperture of the objective onto the entrance slit of the imaging spectrograph (Princeton Instruments, HRS 300, 1200 gr/mm grating) equipped with an air-cooled electron-multiplying CCD (charge-coupled device) camera (512 × 1024 pixels, Andor, iXon 888 Ultra). The resolution of the spectrometer is 0.08 nm.

### Optically pumped lasing experiments

For the CW lasing and PL experiments, the perovskite microcavity samples were pumped by off-resonant excitation using a 405-nm CW solid-state laser (maximum power, 50 mW). For the pulsed lasing and PL experiments, the laser pulses (400 nm; pulse duration: ∼270 fs; repetition rate: 50 kHz) were generated by an optical parametric amplifier (OPA, Orpheus-F, Light Conversion Ltd.) pumped by a 1030-nm Yb^+^:YAG laser (Pharos, Light Conversion Ltd.). The excitation beam was directed through a tunable neutral density filter (Thorlabs, NDC-50C-2M-B). A small fraction of the beam was directed onto a photodiode (Thorlabs, DET110) for pump power monitoring. The rest of the beam was directed into an inverted microscope and then focused onto the sample with a spot size of ~18 μm through a 50× objective. To characterize the intensity profile of the excitation spot, the linear intensity profile of the spot was fitted using the Gaussian function, and the FWHM was determined numerically. The real-space beam profiles were captured by a high-resolution beam profiler CCD camera (Thorlabs, BC207VIS). The polarization properties of the emission were characterized based on the emission intensities measured through a rotatable linear-polarizer plate. The pump intensities reported in this work are intensities of the incident beams (without any corrections for reflection and transmission losses).

### First-order coherence *g*^1^(τ) measurements

Emission from the microcavities was collimated by the microscope objective and then split into two paths in a custom-built Michelson interferometer with a nonpolarizing cube beam splitter. The signals coming from both arms were directed into a beam profiler (Thorlabs, BC207VIS) to obtain the interference pattern. We then measured *g*^1^(τ) by varying the delay time τ between the two arms of the interferometer.

### Fabrication and measurements of the indirectly injected PeLC

The PeLC was constructed by directly attaching a single-crystal perovskite microcavity onto a commercial GaN LED (peak wavelength: 410 nm; maximum power: 3 W) using UV epoxy. The GaN LED (as the pump for the perovskite microcavity) was driven by a dc source (Keithley 2400, which can be replaced with alternative dc sources including batteries). The emission from the laser chip was collected from the objective and fiber coupled into a high-resolution spectrometer (Maya 2000, Ocean Optics).

### NMR measurements

High-resolution NMR measurements were carried out using an Agilent DD2 600 MHz NMR spectrometer. For liquid-state ^1^H NMR measurements, perovskite single crystals were dissolved in 0.6 ml of DMSO-*d*_6_ to form a concentration of 25 mg/ml.

## Supplementary Materials

### This PDF file includes:

Supplementary Notes S1 to S4 Figs. S1 to S16 Table S1 References
