# Supplementary material for: Continuous-wave perovskite polariton lasers
Source: Sci Adv. 2025 Jan 10;11(2):eadr8826. doi: 10.1126/sciadv.adr8826 (PMC11721563; doi:10.1126/sciadv.adr8826)
Supplement: Supplementary file 1 — Supplementary Notes S1 to S4 Figs. S1 to S16 Table S1 References [file sciadv.adr8826_sm.pdf]

Science Advances
AAAS

Supplementary Materials for

Continuous-wave perovskite polariton lasers

Chen Zou *et al.*

Corresponding author: Dawei Di, daweidi@zju.edu.cn; Chen Zou, zouchen@zju.edu.cn;
Baodan Zhao, baodanzhao@zju.edu.cn

Sci. Adv. **11**, eadr8826 (2025)
DOI: 10.1126/sciadv.adr8826

This PDF file includes:

Supplementary Notes S1 to S4
Figs. S1 to S16
Table S1
References

Supplementary Note S1. Fitting of absorption spectra using Elliott formula

The absorption spectra (Fig. 1G) can be modelled using the Elliott formula (*45*):

$$\alpha(\hbar\omega) \propto \frac{\mu_{cv}^2}{\hbar\omega}\left[\sum_j \frac{4\pi\sqrt{E_b^3}}{j^3}\delta\left(\hbar\omega - E_j^b\right) + \frac{2\pi\sqrt{E_b}\theta\left(\hbar\omega - E_g\right)}{1-e^{-2\pi\sqrt{\frac{E_b}{\hbar\omega - E_g}}}}\right] \qquad \text{(S1)}$$

where μ_{cv} is the transition dipole moment, $\hbar\omega$ is the photon energy, $\alpha(\hbar\omega)$ is the absorption coefficient. E_g and E_b are the bandgap energy and the exciton binding energy, respectively. δ(x) and θ(x) are the Dirac-delta and the Heaviside step functions, respectively. The first term in the equation describes transitions to bound states with energy $E_j^b = E_g - \frac{E_b}{j^2}$, while the second term refers to continuum states with energies above the bandgap. The experimentally measured absorption spectrum could be fitted by the Elliott formula, yielding E_g=2.361±0.002 eV and E_b=41±1.2 meV.

The absorption spectrum does not simply follow the square root dependence of the density of states on energy (i.e. $\alpha(\hbar\omega) \propto \sqrt{\hbar\omega - E_g}$) as expected for typical band-to-band transitions between uncorrelated electrons and holes. The contribution from exciton absorption can be clearly observed, indicating the presence of excitons in the sample under optical excitation (*45*).

Supplementary Note S2. Calculation of exciton density based on Saha relationship

The Saha relationship, which describes the thermal equilibrium of the correlated and uncorrelated e-h pairs in semiconductor materials, is written as (*15*):

$$x = \left(n + \frac{A}{2}\right) - \sqrt{\left(\frac{A}{2}\right)^2 + An} \quad \text{(S2)}$$

where x and n are exciton density and the total density of excited species respectively, and

$$A = \left(\frac{\sqrt{2\pi m_{\mathrm{ex}} k_{\mathrm{B}} T}}{h}\right)^3 \exp\left(-\frac{E_{\mathrm{b}}}{k_{\mathrm{B}} T}\right) \quad \text{(S3)}$$

where k_{B} and h are the Boltzmann constant and Planck's constant, respectively, and m_{ex} is the effective mass of exciton ($m_{\mathrm{ex}} = 0.1\, m_e$, m_e is the free electron mass). Fig. S6 shows the calculated exciton fraction (x/n) at room temperature (T = 300 K). For $FA_{0.1}MA_{0.9}PbBr_3$ showing an exciton binding energy of ~30-60 meV at room temperature, x/n increases rapidly above n of ~10^{16} cm^{-3} and reaches 50% at n of ~0.8-2.4 × 10^{17} cm^{-3}. This excitation density corresponds to the pump fluence around ~0.55-1.65 μJ cm^{-2}. The first threshold (0.3 μJ cm^{-2}) in our fs pulse pumping experiments corresponds to an exciton fraction of 34%. At excitation intensities corresponding to the polariton lasing regime, excitons and free carriers are both present with the fraction of excitons reaching >50%, which is sufficient for sustaining an exciton population for polariton interactions.

The Mott transition occurs at a critical density correspond to the transformation from an exciton gas to an electron-hole plasma, which occurs at the stage of photon lasing. The Mott density was reported to be in the range of 1.8-4.7×10^{17} cm^{-3} in lead halide perovskites (*15, 50*).

The exciton binding energy in $FA_{0.1}MA_{0.9}PbBr_3$ is slightly larger than the thermal energy at room temperature (26 meV). In this case, correlated and uncorrelated e–h pairs (i.e., excitons and free carriers) are expected to coexist in thermal equilibrium under excitation intensities near which polariton lasing can occur (10^{16}-10^{18} cm^{-3}), according to the Saha equation. The excitation density corresponding to the first threshold (0.3 μJ cm^{-2}) in our pulsed pumping experiments is estimated to be 4.34×10^{16} cm^{-3}.

Supplementary Note S3. Coupled oscillator model fitting for polariton dispersion

3-1. Angular dispersion of uncoupled cavity modes in perovskite microcavities

In a microcavity, as the vertical component of wavevector for photons is quantized, the energy of cavity photon mode is discrete and their dependence on angle θ is expressed as follows (*15*).

$$E_{\mathrm{cav}}(\theta) = E_{\mathrm{cav}}(0)\left(1 - \frac{\sin^2\theta}{n_{\mathrm{eff}}^2}\right)^{-\frac{1}{2}} \quad \text{(S4)}$$

where $E_{\mathrm{cav}}(0)$ is the cavity mode position at zero angle ($k_{\parallel} = 0$), n_{eff} is the effective refractive index. In a microcavity, n_{eff} can be determined by the overlap integral between the electric field distribution function and the material refractive index profile consisting of the perovskite active layer and dielectrics in DBRs (SiO_2 and TiO_2 having refractive indices of 1.46 and 2.49, respectively). The cavity mode observed in fig. S14 can be fitted by taking $n_{\mathrm{eff}} = 1.9$.

3-2. Coupled oscillator model describing polariton dispersion

Coupling between the cavity photon mode and exciton transition dipole moment is often described by the coupled oscillator model using a phenomenological Hamiltonian H shown as follows (*15*).

$$H = \begin{bmatrix} E_{\mathrm{cav}}(\theta) & \hbar\Omega/2 \\ \hbar\Omega/2 & E_{\mathrm{ex}} \end{bmatrix} \quad \text{(S5)}$$

where E_{ex} is the exciton energy and is treated as constant because their dispersion curve is much less sensitive to θ than to E_{cav}. $\hbar\Omega$ is the Rabi-splitting energy that indicates the strength of coupling. The energies of polariton modes are obtained by solving the eigenvalues for H. As a result, the lower polariton energy E_{pol}^{-} can be written as (*15*):

$$E_{\mathrm{pol}}^{-} = \frac{(E_{\mathrm{cav}}+E_{\mathrm{ex}})-\sqrt{(E_{\mathrm{cav}}-E_{\mathrm{ex}})^2+(\hbar\Omega)^2}}{2} \quad \text{(S6)}$$

Note that E_{cav} depends on θ as shown by Eq. (S4). By using Eq. (S6), we have performed a fitting analysis for the experimentally obtained E_{pol}^{-}, as shown in fig. S12. As the exciton energy and the effective refractive index are reliably determined from the experimental data (including absorption spectra and cavity mode dispersion), the only parameters involved in the curve fitting are the cavity mode energy ($E_{\mathrm{cav}}(0)$) and Rabi splitting energy ($\hbar\Omega$). Using this method, we obtain $\hbar\Omega = 115 \pm 5$ meV and $E_{\mathrm{cav}}(0) = 2.292 \pm 0.002\ eV$. The negative detuning is 28 ± 2 meV for the case of Fig. 2D. Alternative detuning and Rabi splitting energies do not yield satisfactory fitting to the experimental data (fig. S12).

Supplementary Note S4. A summary of evidence for polariton lasing

To accurately identify the lasing mechanism, we follow strictly a well-stablished checklist (see table below) for the recognition of polariton lasing (*33,54*).

Characteristics	Polariton laser	Photon laser	Evidence in paper
Threshold corresponds to onset of degeneracy	✓	×	Fig. 2D, Fig. 2E
Emission blueshift above the threshold	✓	×	Fig. 2B, Fig. 3B
Energy-momentum dispersion at lower polariton branch	✓	×	Fig. 2D, Fig. 3D
Carrier density below Mott density	✓	×	Fig. S6

i) The threshold corresponds to the onset of degeneracy (Fig. 2D), where the polaritons start to condense into the ground-state. The occupied state dispersion at the threshold is consistent with Maxwell-Boltzmann distribution (Fig. 2E). However, the threshold of the photon lasing corresponds to the onset of population inversion and the occupied states do not obey such a distribution. In our pulsed pumping experiments (Fig. 3), the first threshold corresponds to the occurrence of polariton lasing and the second threshold corresponds to the onset of photon lasing.

ii) The CW lasing shows a clear blueshift above the threshold (Fig. 2B). This should not occur in the case of photon lasing.

iii) The photon energies of the emission is distributed along the lower polariton branch (Fig. 2D, Fig. 3D, and fig. S11), whereas the photon energies of emission from a photon laser is located at the bottom of the cavity mode (Fig. 3F).

iv) The concentration of polaritons at the threshold is found to be 4.34×10^{16} cm^{-3} (Supplementary Note S2), which is below the Mott density (1.8-4.7×10^{17} cm^{-3}) in metal halide perovskites (fig. S6) (*15, 50, 55*). Photon lasing (emission from e-h plasma) requires the carrier concentration to clearly exceed the Mott density.

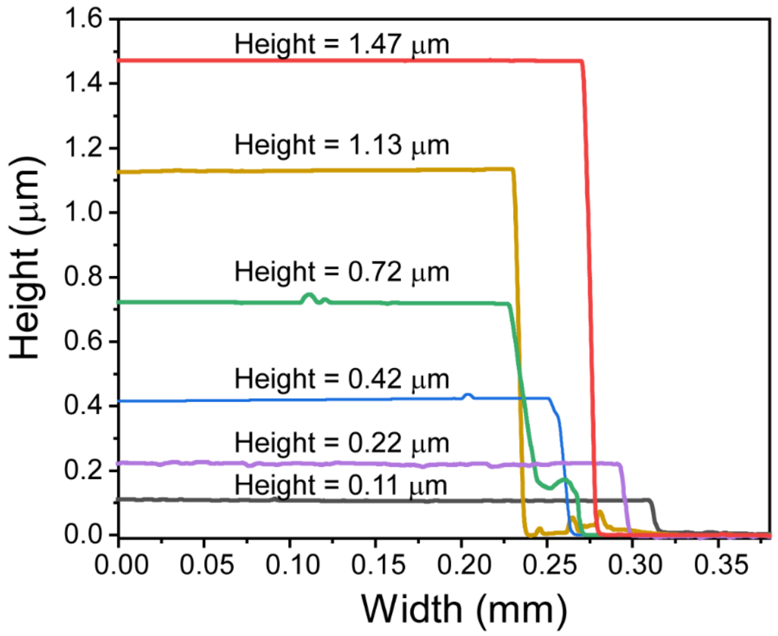

Fig. S1. Surface profiles of perovskite single crystals. The thicknesses (heights) of the single crystals are controlled by the heights of the gold pillar spacers.

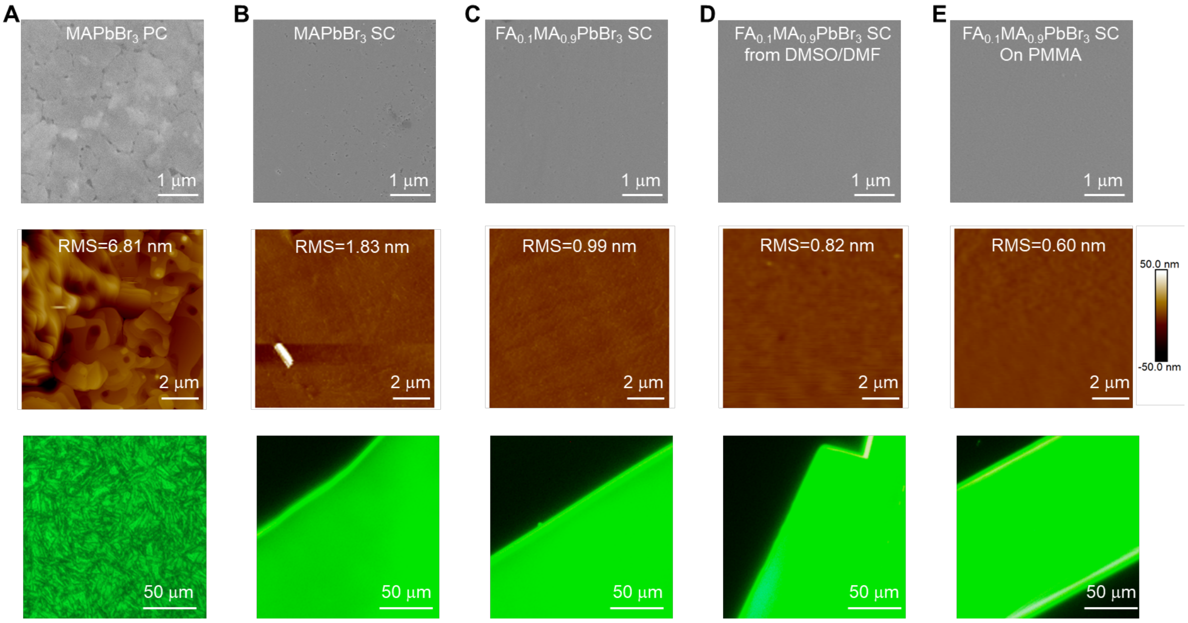

Fig. S2. Surface morphology of perovskite single crystals. SEM (upper panels), AFM (middle panels) and fluorescent (bottom panels) microscopic images of (**A**) $MAPbBr_3$ PC, (**B**) $MAPbBr_3$ SC**,** (**C**) $FA_{0.1}Pb_{0.9}Br_3$ SC, (**D**) $FA_{0.1}Pb_{0.9}Br_3$ SC from DMSO/DMF, (**E**) $FA_{0.1}Pb_{0.9}Br_3$ SC on PMMA. FMPB denotes $FA_{0.1}Pb_{0.9}Br_3$. The root mean square (RMS) roughness of the samples in (**A-E**) are 6.81, 1.83, 0.99, 0.82, 0.60 nm, respectively.

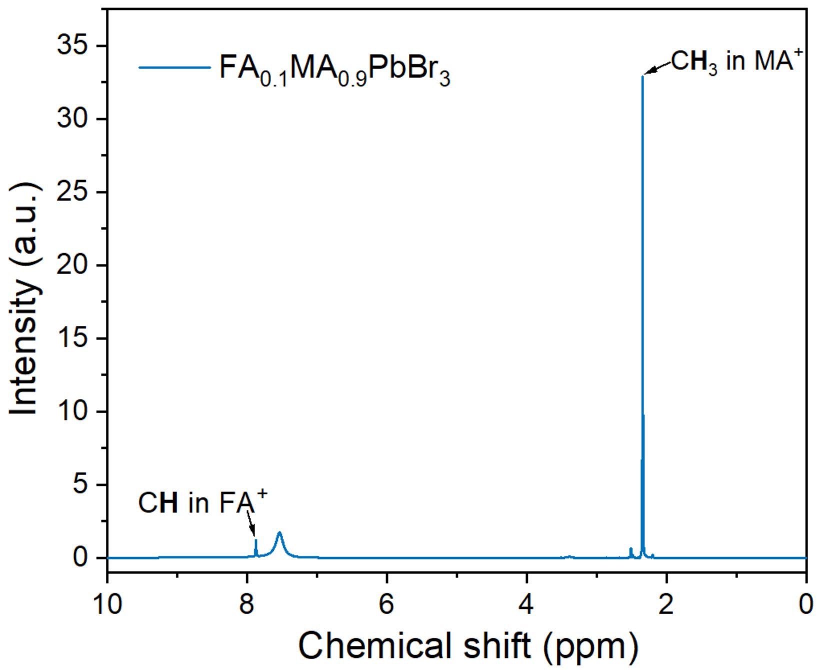

Fig. S3. ¹H NMR spectra of $FA_{0.1}MA_{0.9}PbBr_3$ single crystals. The perovskite single crystals were dissolved in dimethyl sulfoxide-d6 (DMSO-d6) solvent for liquid-state ^{1}H-NMR spectroscopy measurements. The peaks at 7.87 ppm and 2.34 ppm correspond to the hydrogen on CH in FA^+ and CH_3 in MA^+, respectively. By separately integrating the intensities of the two peaks (**C**H and **C**H_3), an intensity ratio of 1:21.4 can be obtained. From this, the molar ratio of FA^+ to MA^+ is found to be 1:7.13. Therefore, the resultant perovskite composition is $FA_{0.12}MA_{0.88}PbBr_3$, which is in close agreement with the intended composition ($FA_{0.1}MA_{0.9}PbBr_3$) determined by the precursor molar ratios.

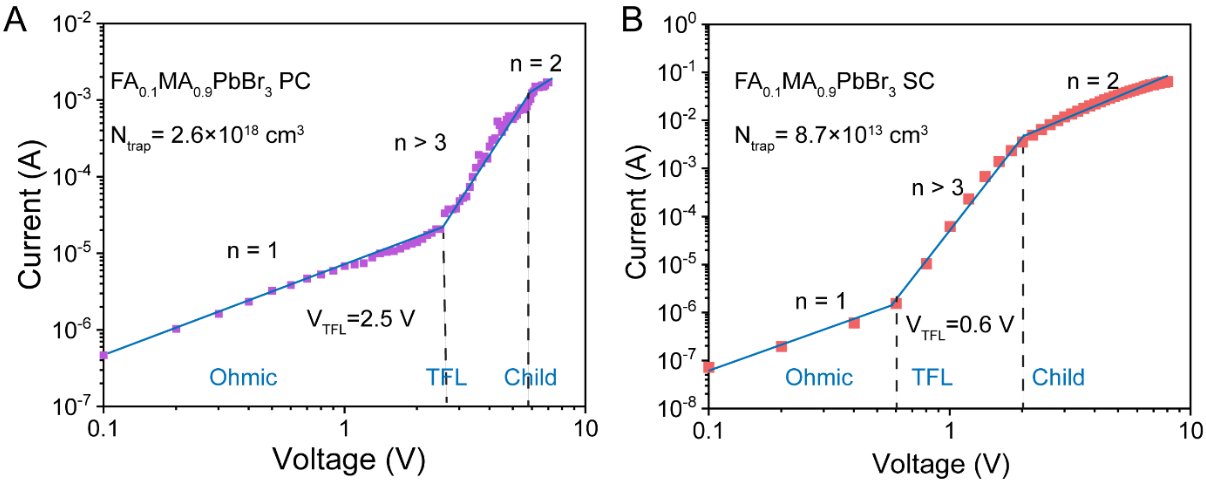

Fig. S4. Current-voltage characteristics of hole-only devices. (**A**) polycrystalline and (**B**) single-crystal $FA_{0.1}MA_{0.9}PbBr_3$. The device structures are ITO/poly-TPD/perovskite/MoO_3/Au. Three distinct regions can be identified: ohmic, trap filled limited, and child regions. The trap density can be extracted from space-charge-limited current (SCLC) model: $N_{trap} = 2\varepsilon_0\varepsilon_r V_{TFL}/eL^2$, where ε_0, ε_r, V_{TFL}, e and L are the vacuum permittivity, the relative dielectric constant, the onset voltage of the TFL region, elementary charge, and the thickness of the perovskite layer, respectively.

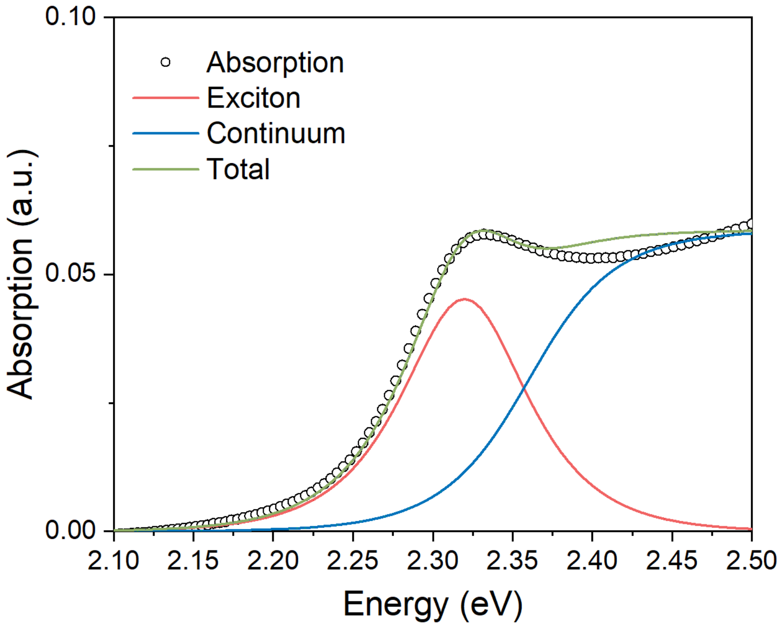

Fig. S5. Absorption spectrum of a $FA_{0.1}MA_{0.9}PbBr_3$ single crystal and the corresponding curve fitting using the Elliott formula. The measured absorption spectrum (black open circles) was fitted according to the Elliott formula (green curve) with contributions from the excitonic (red curve) and continuum (blue curve) band transitions. The experimental absorption spectrum can be fitted by the formula, yielding E_g=2.361±0.002 eV and E_b=41±1.2 meV.

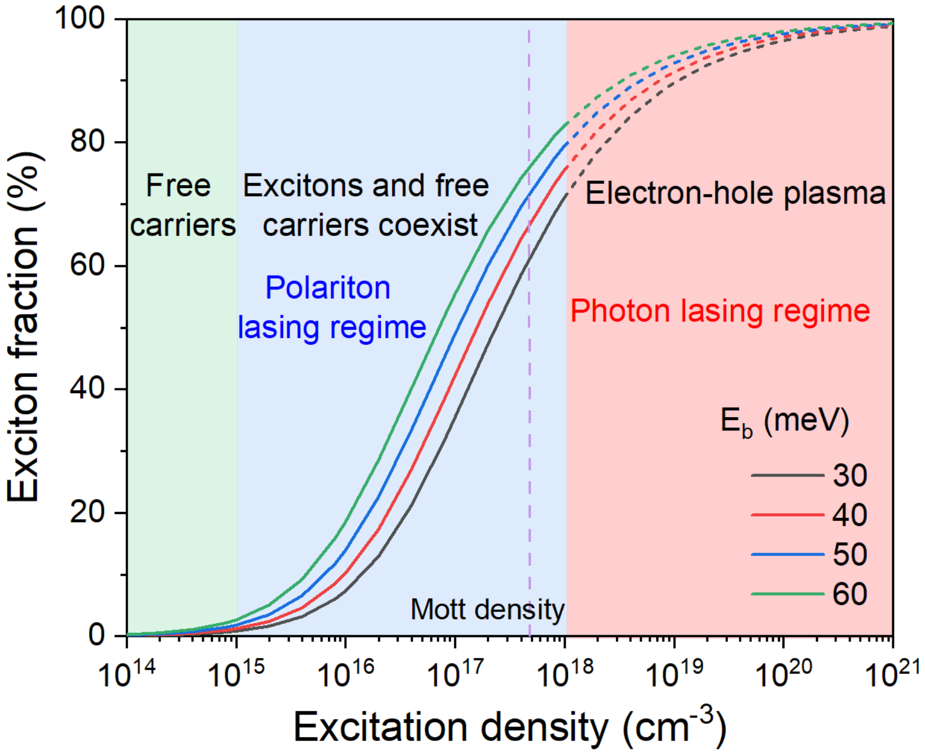

Fig. S6. Exciton fraction x/n as a function of total excitation density (n) calculated from Saha relationship. The vertical dashed line represents the Mott density. The solid curves are exciton fractions under different excitation densities for different binding energies. In the photon lasing regime (dashed curves), the excitons dissociate into electron-hole plasma. For $FA_{0.1}MA_{0.9}PbBr_3$ showing an exciton binding energy of ~30-60 meV at room temperature, x/n increases rapidly above n of ~10^{16} cm^{-3} and reaches 50% at n of ~0.8-2.4 × 10^{17} cm^{-3}. This excitation density corresponds to a pump fluence of around ~0.55-1.65 μJ cm^{-2}. The first threshold (0.3 μJ cm^{-2}) in our pulsed pumping experiments corresponds to an exciton fraction of ~34%.

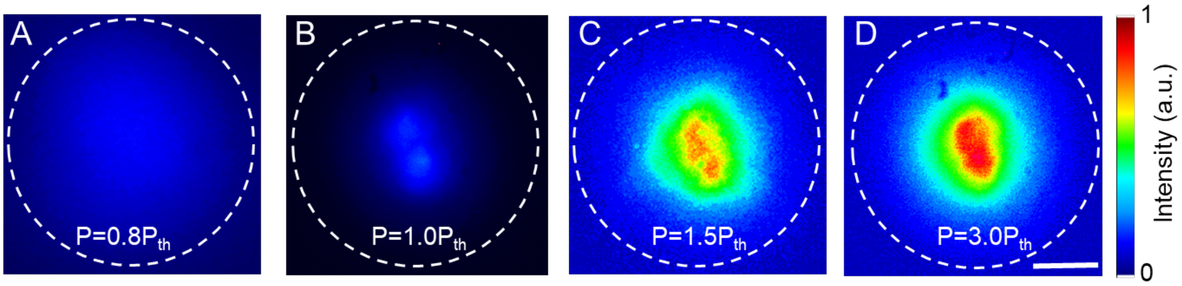

Fig. S7. The real-space images of the emission spot under different CW pump intensities. (**A**) P=0.8P$_{th}$. (**B**) P=1.0P$_{th}$. (**C**) P=1.5P$_{th}$. (**D**) P=3.0P$_{th}$. The dashed circle marks the area of the excitation spot. Scale bar: 5 μm.

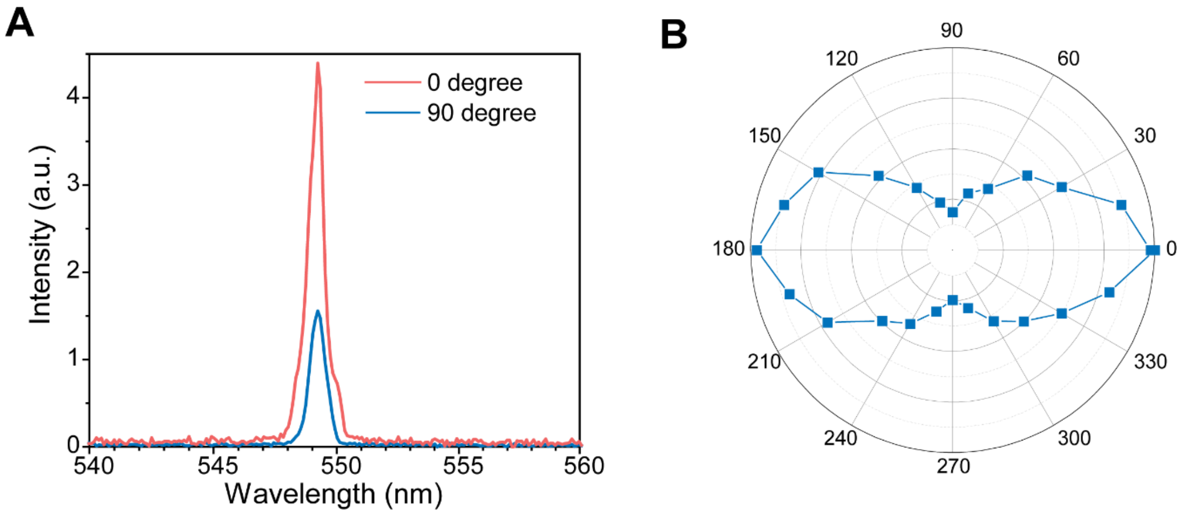

Fig. S8. Polarisation characteristics of CW perovskite polariton laser. (**A**) Lasing spectra at polarisation directions θ = 0° and 90°. (**B**) Polar plot of linear polarisation, showing the intensity of ground-state emission as a function of the polarisation angle. Excitation wavelength: 405 nm; Excitation power: 1.5 W cm^{-2} (above threshold).

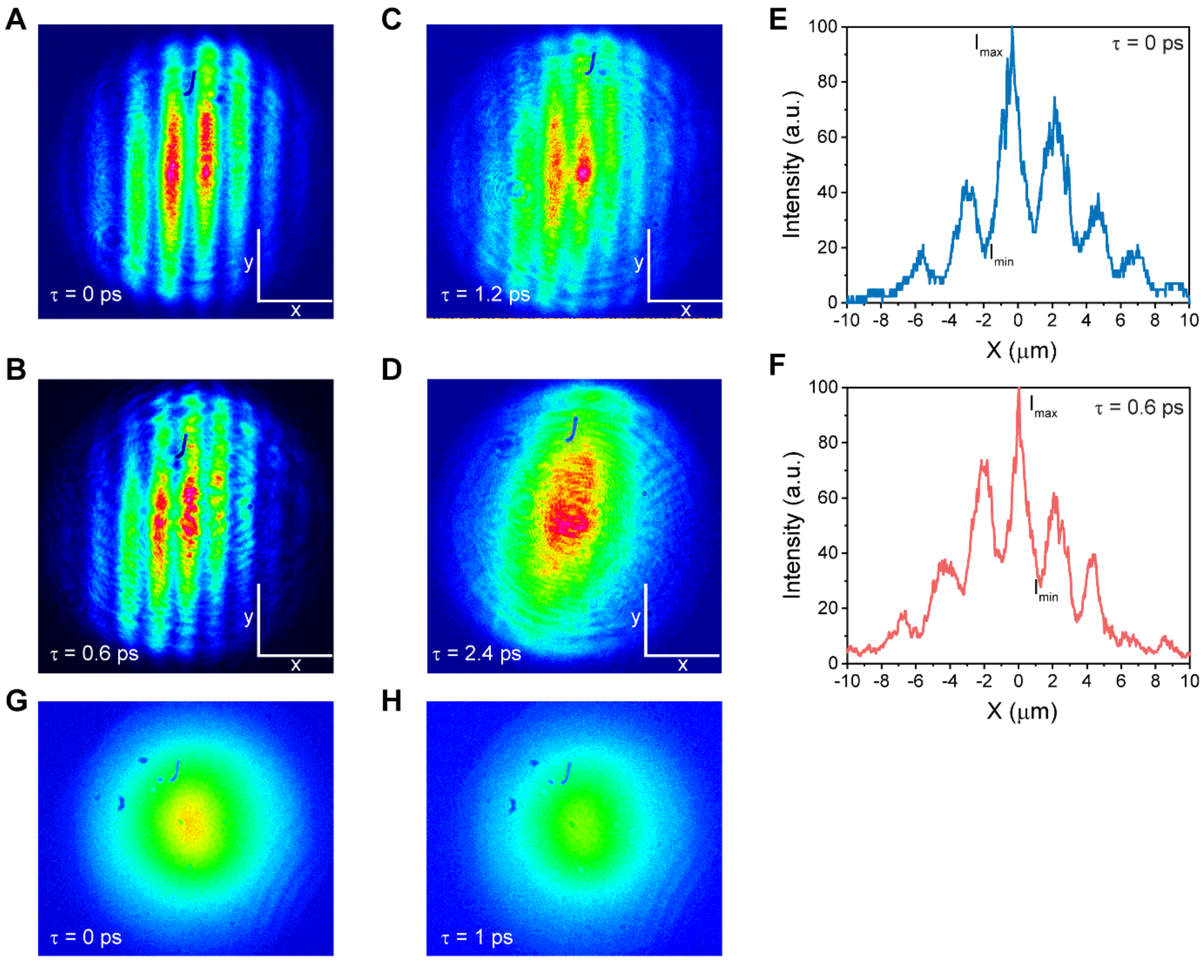

Fig. S9. First-order coherence $g^1(\tau)$ properties of the CW perovskite polariton laser. (**A-D**) The spatial interference image of the polariton laser (above threshold, 5.0P_{th}) at time delay τ = 0 ps **(A)**, τ = 0.6 ps **(B)**, τ = 1.2 ps **(C)** and τ = 2.4 ps **(D)**. The scale bar is 5 μm along x and y axis. (**E**) The intensity profile of interference fringes along x axis obtained from (a), at zero time delay. (**F**) The intensity profile of interference fringes along x axis obtained from (b), by introducing a slightly different time delay (τ = 0.6 ps.), the interference fringes remain clearly visible. The I_{max} and I_{min} represents the maximum and minimum intensity of the center fringe, the first-order coherence could be calculated from $g^1(\tau) = (I_{max} - I_{min})/(I_{max} + I_{min})$. (**G-H**) The spatial interference images of the polariton laser (below threshold, 0.8P_{th}) at time delay τ = 0 ps (**G**), τ = 1 ps (**H**).

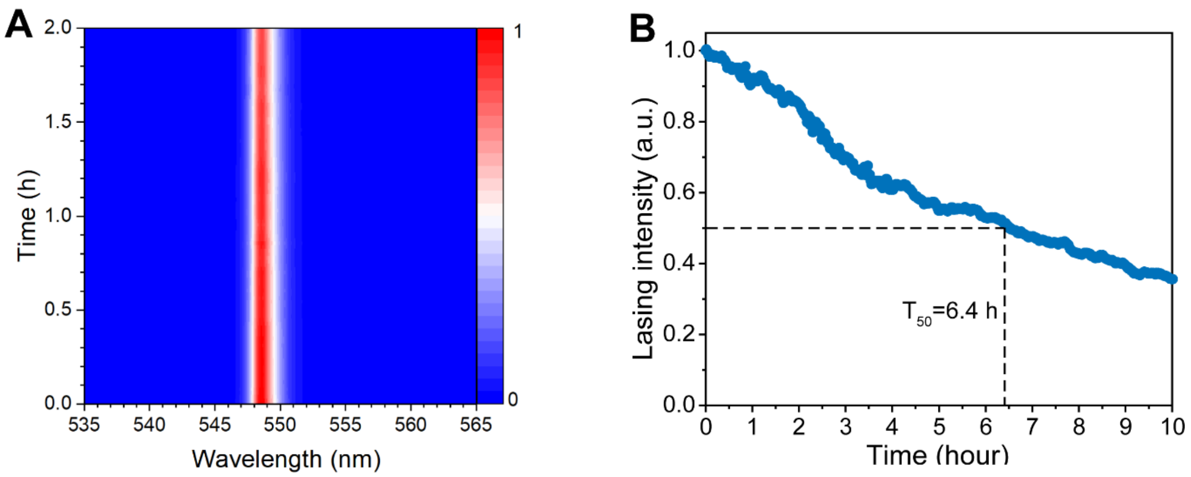

Fig. S10. Stability tests of CW perovskite polariton lasers. (**A**) Evolution of lasing spectra over time. (**B**) Output intensity of a CW perovskite polariton laser as a function of time, showing a T_{50} lifetime (the time required for the output intensity to reduce to 50% of its initial value) of 6.4 hour.

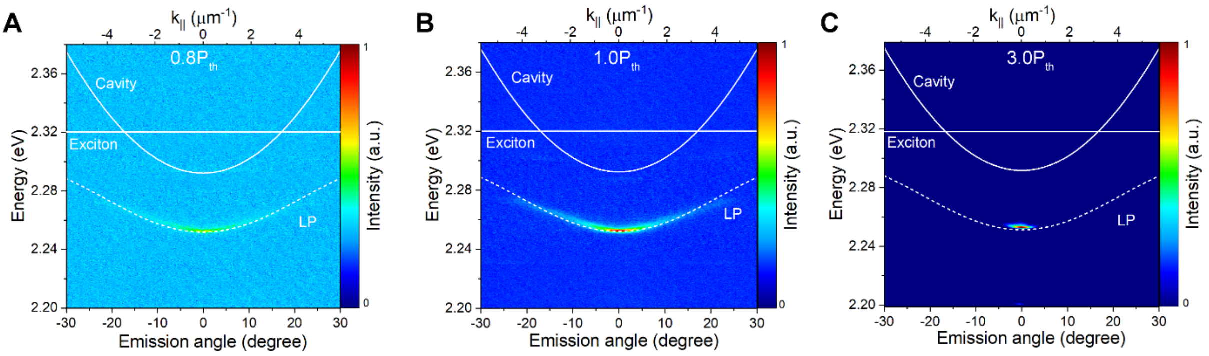

Fig. S11. Angle-resolved PL spectra of a single-crystal perovskite microcavity under CW pumping. The CW pump intensities are (**A**) $0.8P_{th}$ (**B**) $1.0P_{th}$ and (**C**) $3P_{th}$. P_{th} represents the threshold of the CW perovskite polariton laser.

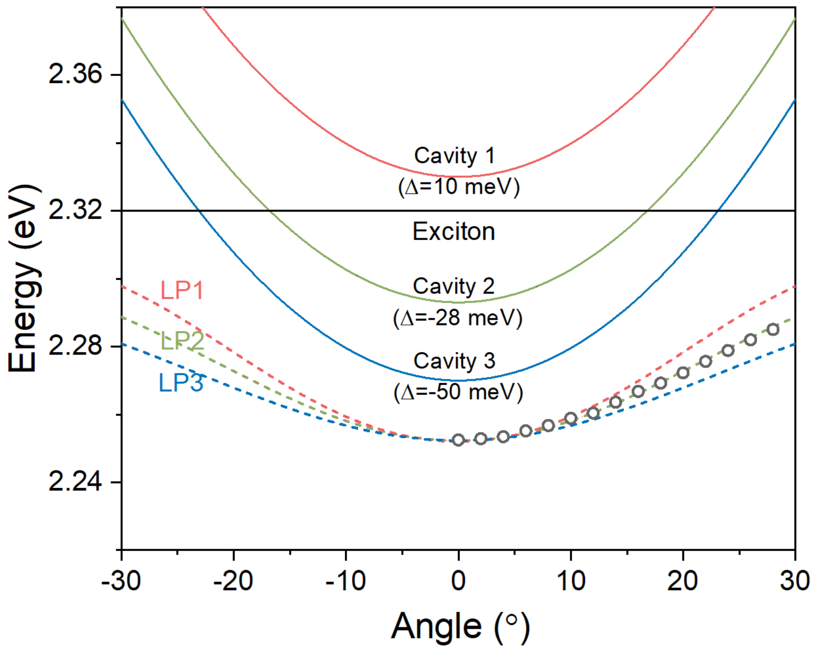

Fig. S12. Fitting analysis of angle-dependent polariton emission using a coupled oscillator model. Black open circles represent the PL peak energies extracted from Fig. 2D. The black line corresponds to the exciton energy (E_{ex} ~ 2.32 eV). The coloured curves represent uncoupled cavity photon mode (E_{cav}) with n_{eff} = 1.9, with different cavity mode energies (E_{cav} (0) = 2.330, 2.292 and 2.270 eV, corresponding to detuning energies of Δ = 10, -28, and -50 meV, respectively). The coloured dashed curves are the dispersions of the corresponding lower polariton branches with Rabi splitting energies ($\hbar\Omega$) of 145, 115 and 70 meV. We note that only the green dashed curve agrees closely with the experimental data (black open circles). Alternative detuning and Rabi splitting energies do not yield satisfactory fitting to the experimental data. The results are shown in the table below.

**Curve colour**	$E_{cav}(0)$ **(eV)**	**Δ (meV)**	$\hbar\Omega$ **(meV)**	**Deviation of fitting (meV)**
Red	2.330	10	145	32
Green	2.292	-28	115	1.6
Blue	2.270	-50	70	37

In this analysis, $\hbar\Omega$ and E_{cav} (0) are the only fitting parameters. The fitting results with uncertainties are: $\hbar\Omega = 115 \pm 5$ meV and $E_{cav}(0) = 2.292 \pm 0.002\ eV$. Other details of the fitting are shown below.

**Parameter**	**Value**	**Uncertainty**
Exciton energy (E_{ex})	2.32 eV	0.003 eV
Effective refractive index (n_{eff})	1.9	0.04
Cavity mode energy ($E_{cav}(0)$)	2.292 eV	0.002 eV
Detuning (Δ)	-0.028 eV	0.002 eV
Rabi Splitting ($\hbar\Omega$)	0.115 eV	0.005 eV

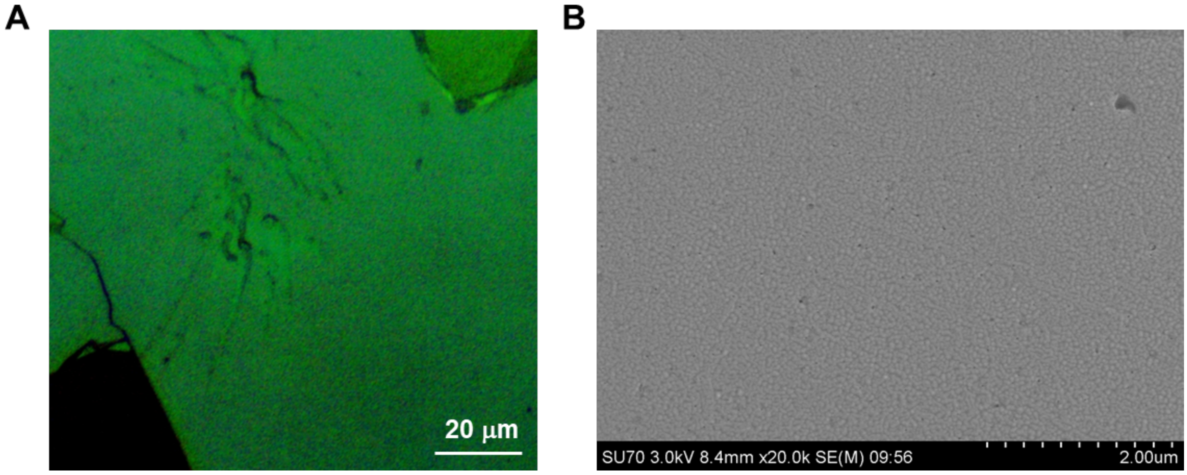

Fig. S13. Perovskite single crystals with lower optical quality. The samples were prepared by rapidly evaporating the precursor solution at 120 °C for 10 min. **a**, Fluorescence image of the lower-quality perovskite single crystals. **b**, The SEM image of the sample, showing a relatively rough surface.

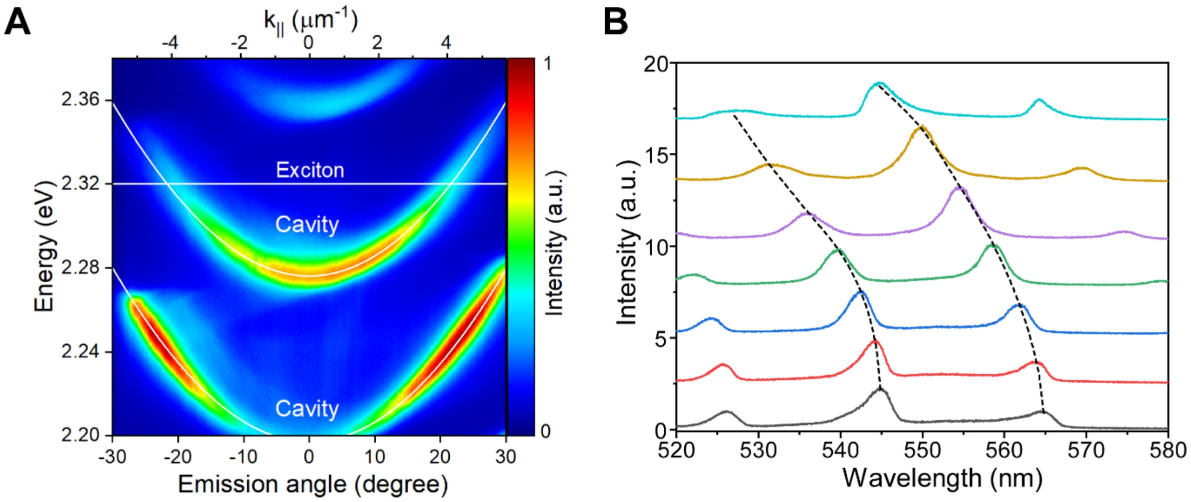

Fig. S14. Performance of a weakly coupled single-crystal perovskite microcavity under CW pumping. (**A**) Angle-resolved PL spectra at a CW pump intensity of 5.7 W cm^{-2}. Only the cavity mode is observed, indicating the sample is in a weakly coupled regime. (**B**) The PL spectra of the sample at emission angles from 0 to 30°. The cavity mode has a distinct "parabolic" shape and can cross over the exciton mode, while the polariton branch is more flattened at larger angles and always below the exciton mode.

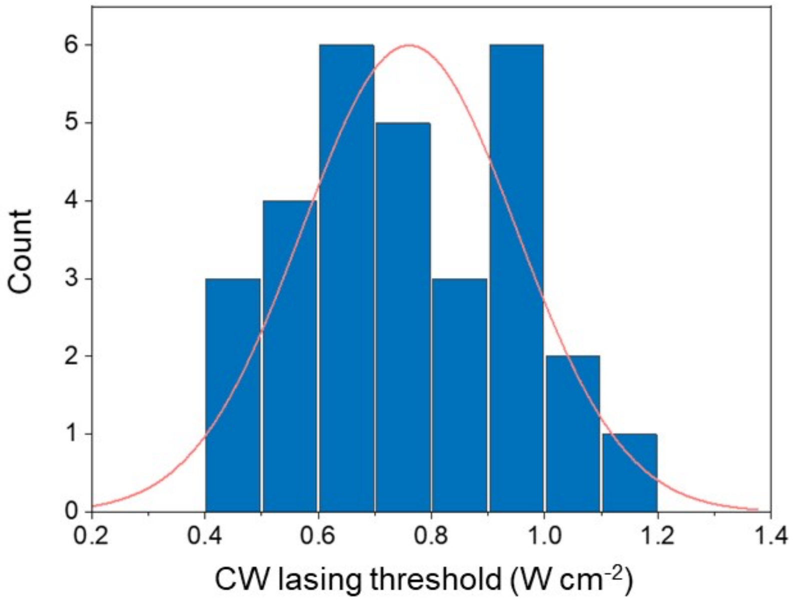

Fig. S15. Histogram of CW lasing thresholds of our perovskite polariton lasers.

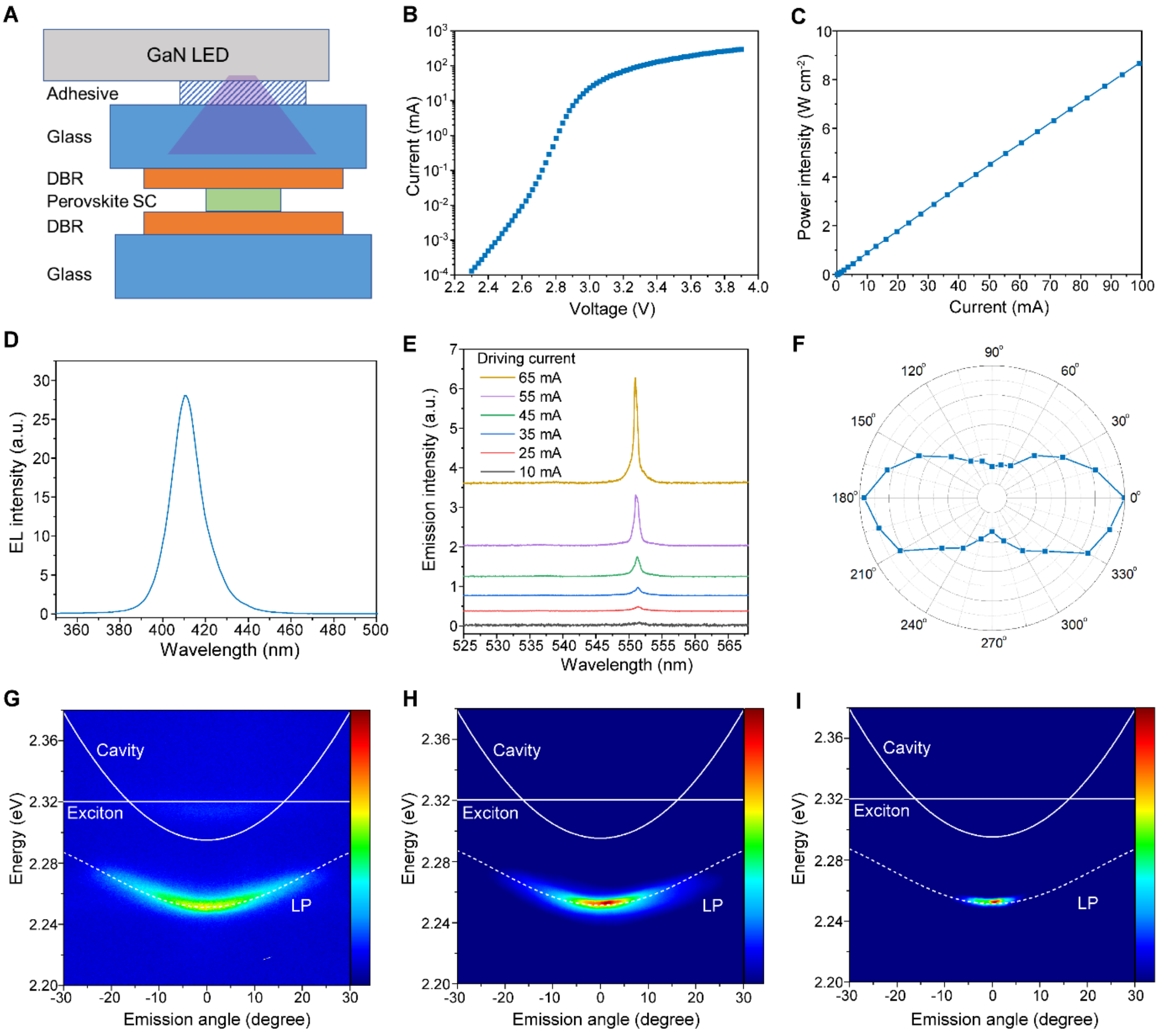

Fig. S16. Additional characterization of the PeLC. (**A**) Schematic configuration of the PeLC. (**B**) Current-voltage characteristics of the GaN LED subunit. (**C**) Power intensity-current characteristics of the GaN LED subunit. (**D**) EL spectrum of the GaN LED subunit. (**E**) Emission spectra of the PeLC at different driving currents. (**F**) Polar plot of linear polarization, showing the output intensity of the PeLC as a function of the polarization angle at a driving current of 65 mA (above threshold). (**G-I**) Angle-resolved EL spectra of the PeLC at driving currents of (**G**) 25 mA (below threshold), (**H**) 45 mA (around threshold), and (**I**) 65 mA (above threshold).

Table S1. Lasing thresholds of optically pumped semiconductor lasers. The pump pulse durations of the quasi-CW lasers are listed under 'pumping condition'. 'RT' stands for room temperature.

Laser type	Gain medium material	Threshold (W cm^{-2})	Pumping condition	Temperature	Ref.
III-V	GaAs	1.45×10^4	CW	10 K	(*28*)
III-V	InAs	1592	CW	RT	(*56*)
III-V	InGaN/GaN	1000	CW	RT	(*57*)
III-V	InP/InGaAs	200	CW	RT	(*29*)
III-V	InAs/GaAs	52	CW	RT	(*58*)
III-V	InAs/GaAs	12.2	CW	RT	(*27*)
II-VI	CdSe/Zn(Cd)Se	4000	CW	10 K	(*59*)
II-VI	CdSe/Zn(Cd)Se	800	CW	RT	(*60*)
II-VI	CdTe	500	CW	19 K	(*31*)
Perovskite	$MAPbI_3$	1.7×10^4	CW	102 K	(*13*)
Perovskite	$CsPbBr_3$	6000	CW	77 K	(*61*)
Perovskite	$CsPbBr_3$	2600	CW	78 K	(*62*)
Perovskite	$Cs_{0.1}(MA_{0.17}FA_{0.83})_{0.9}Pb_{0.84}(I_{0.84}Br_{0.16})_{2.68}$	387	CW	80 K	(*63*)
Perovskite	$(NMA)FAPbBr_3$	45	CW	RT	(*12*)
Perovskite	$MAPbBr_3$	15	CW	RT	(*25*)
Perovskite	$MAPbI_3$	13	CW	RT	(*24*)
**Perovskite**	**$FA_{0.1}MA_{0.9}PbBr_3$**	**0.4**	**CW**	**RT**	**This work**
CQD	CdSe	8000	CW	RT	(*6*)
CQD	CdSe	440	CW	RT	(*9*)
CQD	CdSe	214	CW	RT	(*8*)
Organic	EHCz:heptafluorene	2750	Pulsed (quasi-CW), 0.8 ns	RT	(*64*)
Organic	BBEHP-PPV	770	Pulsed (quasi-CW), 4 ns	RT	(*65*)
Organic	BSBCz:DCNP	72	Pulsed (quasi-CW), 10 ms	RT	(*66*)
Organic	Y80F8:20F5	30	Pulsed (quasi-CW), 12 ns	RT	(*67*)
Organic	BSBCZ:CBP	5	Pulsed (quasi-CW), 10 μs	RT	(4)
Oxide	ZnO	1.7×10^5	Pulsed (quasi-CW), 10 ns	RT	(*68*)
Oxide	ZnO	4×10^4	Pulsed (quasi-CW), 3 ns	RT	(*69*)
Oxide	ZnO	3.18×10^4	Pulsed (quasi-CW), 0.5 ns	RT	(*52*)
Oxide	ZnO/ZnMgO	6000	Pulsed (quasi-CW), 6 ns	RT	(*70*)

REFERENCES AND NOTES

1. N. Tessler, G. J. Denton, R. H. Friend, Lasing from conjugated-polymer microcavities. *Nature* **382**, 695–697 (1996).

2. F. Hide, M. A. Díaz-García, B. J. Schwartz, M. R. Andersson, Q. Pei, A. J. Heeger, Semiconducting polymers: A new class of solid-state laser materials. *Science* **273**, 1833–1836 (1996).

3. S. Kéna-Cohen, S. R. Forrest, Room-temperature polariton lasing in an organic single-crystal microcavity. *Nat. Photon.* **4**, 371–375 (2010).

4. A. S. D. Sandanayaka, T. Matsushima, F. Bencheikh, K. Yoshida, M. Inoue, T. Fujihara, K. Goushi, J.-C. Ribierre, C. Adachi, Toward continuous-wave operation of organic semiconductor lasers. *Sci. Adv.* **3**, e1602570 (2017).

5. I. D. W. Samuel, G. A. Turnbull, Organic semiconductor lasers. *Chem. Rev.* **107**, 1272–1295 (2007).

6. F. Fan, O. Voznyy, R. P. Sabatini, K. T. Bicanic, M. M. Adachi, J. R. McBride, K. R. Reid, Y. S. Park, X. Li, A. Jain, R. Quintero-Bermudez, M. Saravanapavanantham, M. Liu, M. Korkusinski, P. Hawrylak, V. I. Klimov, S. J. Rosenthal, S. Hoogland, E. H. Sargent, Continuous-wave lasing in colloidal quantum dot solids enabled by facet-selective epitaxy. *Nature* **544**, 75–79 (2017).

7. H. Jung, N. Ahn, V. I. Klimov, Prospects and challenges of colloidal quantum dot laser diodes. *Nat. Photon.* **15**, 643–655 (2021).

8. Z. Yang, M. Pelton, I. Fedin, D. V. Talapin, E. Waks, A room temperature continuous-wave nanolaser using colloidal quantum wells. *Nat. Commun.* **8**, 143 (2017).

9. J. Q. Grim, S. Christodoulou, F. Di Stasio, R. Krahne, R. Cingolani, L. Manna, I. Moreels, Continuous-wave biexciton lasing at room temperature using solution-processed quantum wells. *Nat. Nanotechnol.* **9**, 891–895 (2014).

10. F. Deschler, M. Price, S. Pathak, L. E. Klintberg, D. D. Jarausch, R. Higler, S. Huttner, T. Leijtens, S. D. Stranks, H. J. Snaith, M. Atature, R. T. Phillips, R. H. Friend, High photoluminescence efficiency and optically pumped lasing in solution-processed mixed halide perovskite semiconductors. *J. Phys. Chem. Lett.* **5**, 1421–1426 (2014).

11. G. Xing, N. Mathews, S. S. Lim, N. Yantara, X. Liu, D. Sabba, M. Gratzel, S. Mhaisalkar, T. C. Sum, Low-temperature solution-processed wavelength-tunable perovskites for lasing. *Nat. Mater.* **13**, 476–480 (2014).

12. C. Qin, A. S. D. Sandanayaka, C. Zhao, T. Matsushima, D. Zhang, T. Fujihara, C. Adachi, Stable room-temperature continuous-wave lasing in quasi-2D perovskite films. *Nature* **585**, 53–57 (2020).

13. Y. Jia, R. A. Kerner, A. J. Grede, B. P. Rand, N. C. Giebink, Continuous-wave lasing in an organic–inorganic lead halide perovskite semiconductor. *Nat. Photon.* **11**, 784–788 (2017).

14. R. Su, A. Fieramosca, Q. Zhang, H. S. Nguyen, E. Deleporte, Z. Chen, D. Sanvitto, T. C. H. Liew, Q. Xiong, Perovskite semiconductors for room-temperature exciton-polaritonics. *Nat. Mater.* **20**, 1315–1324 (2021).

15. S. Enomoto, T. Tagami, Y. Ueda, Y. Moriyama, K. Fujiwara, S. Takahashi, K. Yamashita, Drastic transitions of excited state and coupling regime in all-inorganic perovskite microcavities characterized by exciton/plasmon hybrid natures. *Light Sci. Appl.* **11**, 8 (2022).

16. W. Du, S. Zhang, Q. Zhang, X. Liu, Recent progress of strong exciton-photon coupling in lead halide perovskites. *Adv. Mater.* **31**, e1804894 (2019).

17. R. Su, C. Diederichs, J. Wang, T. C. H. Liew, J. Zhao, S. Liu, W. Xu, Z. Chen, Q. Xiong, Room-temperature polariton lasing in all-inorganic perovskite nanoplatelets. *Nano Lett.* **17**, 3982–3988 (2017).

18. Y. Cao, N. Wang, H. Tian, J. Guo, Y. Wei, H. Chen, Y. Miao, W. Zou, K. Pan, Y. He, H. Cao, Y. Ke, M. Xu, Y. Wang, M. Yang, K. Du, Z. Fu, D. Kong, D. Dai, Y. Jin, G. Li, H. Li, Q. Peng, J.

Wang, W. Huang, Perovskite light-emitting diodes based on spontaneously formed submicrometre-scale structures. *Nature* **562**, 249–253 (2018).

19. D. Ma, K. Lin, Y. Dong, H. Choubisa, A. H. Proppe, D. Wu, Y.-K. Wang, B. Chen, P. Li, J. Z. Fan, Distribution control enables efficient reduced-dimensional perovskite LEDs. *Nature* **599**, 594–598 (2021).

20. B. Guo, R. Lai, S. Jiang, L. Zhou, Z. Ren, Y. Lian, P. Li, X. Cao, S. Xing, Y. Wang, W. Li, C. Zou, M. Chen, Z. Hong, C. Li, B. Zhao, D. Di, Ultrastable near-infrared perovskite light-emitting diodes. *Nat. Photon.* **16**, 637–643 (2022).

21. J. S. Kim, J.-M. Heo, G.-S. Park, S.-J. Woo, C. Cho, H. J. Yun, D.-H. Kim, J. Park, S.-C. Lee, S.-H. Park, E. Yoon, N. C. Greenham, T.-W. Lee, Ultra-bright, efficient and stable perovskite light-emitting diodes. *Nature* **611**, 688–694 (2022).

22. Y. Hassan, J. H. Park, M. L. Crawford, A. Sadhanala, J. Lee, J. C. Sadighian, E. Mosconi, R. Shivanna, E. Radicchi, M. Jeong, C. Yang, H. Choi, S. H. Park, M. H. Song, F. De Angelis, C. Y. Wong, R. H. Friend, B. R. Lee, H. J. Snaith, Ligand-engineered bandgap stability in mixed-halide perovskite LEDs. *Nature* **591**, 72–77 (2021).

23. S. Hou, M. K. Gangishetty, Q. Quan, D. N. Congreve, Efficient blue and white perovskite light-emitting diodes via manganese doping. *Joule* **2**, 2421–2433 (2018).

24. Z. Li, J. Moon, A. Gharajeh, R. Haroldson, R. Hawkins, W. Hu, A. Zakhidov, Q. Gu, Room-temperature continuous-wave operation of organometal halide perovskite lasers. *ACS Nano* **12**, 10968–10976 (2018).

25. L. Wang, L. Meng, L. Chen, S. Huang, X. Wu, G. Dai, L. Deng, J. Han, B. Zou, C. Zhang, H. Zhong, Ultralow-threshold and color-tunable continuous-wave lasing at room-temperature from in situ fabricated perovskite quantum dots. *J. Phys. Chem. Lett.* **10**, 3248–3253 (2019).

26. C. Tian, T. Guo, S. Zhao, W. Zhai, C. Ge, G. Ran, Low-threshold room-temperature continuous-wave optical lasing of single-crystalline perovskite in a distributed reflector microcavity. *RSC Adv.* **9**, 35984–35989 (2019).

27. T. Zhou, M. Tang, G. Xiang, B. Xiang, S. Hark, M. Martin, T. Baron, S. Pan, J.-S. Park, Z. Liu, S. Chen, Z. Zhang, H. Liu, Continuous-wave quantum dot photonic crystal lasers grown on on-axis Si (001). *Nat. Commun.* **11**, 977 (2020).

28. D. Bajoni, P. Senellart, E. Wertz, I. Sagnes, A. Miard, A. Lemaître, J. Bloch, Polariton laser using single micropillar GaAs-GaAlAs semiconductor cavities. *Phys. Rev. Lett.* **100**, 047401 (2008).

29. F. Lu, I. Bhattacharya, H. Sun, T.-T. D. Tran, K. W. Ng, G. N. Malheiros-Silveira, C. Chang-Hasnain, Nanopillar quantum well lasers directly grown on silicon and emitting at silicon-transparent wavelengths. *Optica* **4**, 717–723 (2017).

30. I. D. W. Samuel, E. B. Namdas, G. A. Turnbull, How to recognize lasing. *Nat. Photon.* **3**, 546–549 (2009).

31. J. Kasprzak, M. Richard, S. Kundermann, A. Baas, P. Jeambrun, J. M. J. Keeling, F. M. Marchetti, M. H. Szymanska, R. Andre, J. L. Staehli, V. Savona, P. B. Littlewood, B. Deveaud, L. S. Dang, Bose-Einstein condensation of exciton polaritons. *Nature* **443**, 409–414 (2006).

32. C. Schneider, A. Rahimi-Iman, N. Y. Kim, J. Fischer, I. G. Savenko, M. Amthor, M. Lermer, A. Wolf, L. Worschech, V. D. Kulakovskii, I. A. Shelykh, M. Kamp, S. Reitzenstein, A. Forchel, Y. Yamamoto, S. Hofling, An electrically pumped polariton laser. *Nature* **497**, 348–352 (2013).

33. T. Byrnes, N. Y. Kim, Y. Yamamoto, Exciton–polariton condensates. *Nat. Phys.* **10**, 803–813 (2014).

34. D. Sanvitto, S. Kéna-Cohen, The road towards polaritonic devices. *Nat. Mater.* **15**, 1061–1073 (2016).

35. C. Anton-Solanas, M. Waldherr, M. Klaas, H. Suchomel, T. H. Harder, H. Cai, E. Sedov, S. Klembt, A. V. Kavokin, S. Tongay, K. Watanabe, T. Taniguchi, S. Höfling, C. Schneider, Bosonic condensation of exciton-polaritons in an atomically thin crystal. *Nat. Mater.* **20**, 1233–1239 (2021).

36. L. V. Butov, A polariton laser. *Nature* **447**, 540–541 (2007).

37. J. Tang, J. Zhang, Y. Lv, H. Wang, F. F. Xu, C. Zhang, L. Sun, J. Yao, Y. S. Zhao, Room temperature exciton–polariton Bose–Einstein condensation in organic single-crystal microribbon cavities. *Nat. Commun.* **12**, 3265 (2021).

38. R. Su, S. Ghosh, J. Wang, S. Liu, C. Diederichs, T. C. H. Liew, Q. Xiong, Observation of exciton polariton condensation in a perovskite lattice at room temperature. *Nat. Phys.* **16**, 301–306 (2020).

39. M. Saba, T. A. Pasquini, C. Sanner, Y. Shin, W. Ketterle, D. E. Pritchard, Light scattering to determine the relative phase of two Bose-Einstein condensates. *Science* **307**, 1945–1948 (2005).

40. W. Chen, Z. Huang, H. Yao, Y. Liu, Y. Zhang, Z. Li, H. Zhou, P. Xiao, T. Chen, H. Sun, J. Huang, Z. Xiao, Highly bright and stable single-crystal perovskite light-emitting diodes. *Nat. Photon.* **17**, 401–407 (2023).

41. R. Tao, K. Peng, L. Haeberle, Q. Li, D. Jin, G. R. Fleming, S. Kena-Cohen, X. Zhang, W. Bao, Halide perovskites enable polaritonic XY spin Hamiltonian at room temperature. *Nat. Mater.* **21**, 761–766 (2022).

42. K. Peng, R. Tao, L. Haeberlé, Q. Li, D. Jin, G. R. Fleming, S. Kéna-Cohen, X. Zhang, W. Bao, Room-temperature polariton quantum fluids in halide perovskites. *Nat. Commun.* **13**, 7388 (2022).

43. J. Feng, J. Wang, A. Fieramosca, R. Bao, J. Zhao, R. Su, Y. Peng, T. C. H. Liew, D. Sanvitto, Q. Xiong, All-optical switching based on interacting exciton polaritons in self-assembled perovskite microwires. *Sci. Adv.* **7**, eabj6627 (2021).

44. J. W. Lee, S. Tan, S. I. Seok, Y. Yang, N. G. Park, Rethinking the A cation in halide perovskites. *Science* **375**, eabj1186 (2022).

45. M. Saba, M. Cadelano, D. Marongiu, F. Chen, V. Sarritzu, N. Sestu, C. Figus, M. Aresti, R. Piras, A. Geddo Lehmann, C. Cannas, A. Musinu, F. Quochi, A. Mura, G. Bongiovanni, Correlated electron-hole plasma in organometal perovskites. *Nat. Commun.* **5**, 5049 (2014).

46. S. Chen, C. Zhang, J. Lee, J. Han, A. Nurmikko, High-Q, low-threshold monolithic perovskite thin-film vertical-cavity lasers. *Adv. Mater.* **29**, 1604781 (2017).

47. N. H. M. Dang, D. Gerace, E. Drouard, G. Trippé-Allard, F. Lédée, R. Mazurczyk, E. Deleporte, C. Seassal, H. S. Nguyen, Tailoring dispersion of room-temperature exciton-polaritons with perovskite-based subwavelength metasurfaces. *Nano Lett.* **20**, 2113–2119 (2020).

48. K. Yoshida, J. Gong, A. L. Kanibolotsky, P. J. Skabara, G. A. Turnbull, I. D. W. Samuel, Electrically driven organic laser using integrated OLED pumping. *Nature* **621**, 746–752 (2023).

49. K. Fujiwara, S. Zhang, S. Takahashi, L. Ni, A. Rao, K. Yamashita, Excitation dynamics in layered lead halide perovskite crystal slabs and microcavities. *ACS Photonics* **7**, 845–852 (2020).

50. A. P. Schlaus, M. S. Spencer, K. Miyata, F. Liu, X. Wang, I. Datta, M. Lipson, A. Pan, X.-Y. Zhu, How lasing happens in $CsPbBr_3$ perovskite nanowires. *Nat. Commun.* **10**, 265 (2019).

51. J. J. Baumberg, A. V. Kavokin, S. Christopoulos, A. J. D. Grundy, R. Butte, G. Christmann, D. D. Solnyshkov, G. Malpuech, G. B. H. von Hogersthal, E. Feltin, J.-F. Carlin, N. Grandjean, Spontaneous polarization buildup in a room-temperature polariton laser. *Phys. Rev. Lett.* **101**, 136409 (2008).

52. T. C. Lu, Y. Y. Lai, Y. P. Lan, S. W. Huang, J. R. Chen, Y. C. Wu, W. F. Hsieh, H. Deng, Room temperature polariton lasing vs. photon lasing in a ZnO-based hybrid microcavity. *Opt. Express* **20**, 5530–5537 (2012).

53. S. Azzini, D. Gerace, M. Galli, I. Sagnes, R. Braive, A. Lemaître, J. Bloch, D. Bajoni, Ultra-low threshold polariton lasing in photonic crystal cavities. *Appl. Phys. Lett.* **99**, 111106 (2011).

54. H. Deng, G. Weihs, D. Snoke, J. Bloch, Y. Yamamoto, Polariton lasing vs. photon lasing in a semiconductor microcavity. *Proc. Natl. Acad. Sci. U.S.A.* **100**, 15318–15323 (2003).

55. J. S. Manser, J. A. Christians, P. V. Kamat, Intriguing optoelectronic properties of metal halide perovskites. *Chem. Rev.* **116**, 12956–13008 (2016).

56. Y. Wan, Q. Li, A. Y. Liu, A. C. Gossard, J. E. Bowers, E. L. Hu, K. M. Lau, Optically pumped 1.3 μm room-temperature InAs quantum-dot micro-disk lasers directly grown on (001) silicon. *Opt. Lett.* **41**, 1664–1667 (2016).

57. M. Athanasiou, R. Smith, B. Liu, T. Wang, Room temperature continuous-wave green lasing from an InGaN microdisk on silicon. *Sci. Rep.* **4**, 7250 (2014).

58. H. Zhong, Y. Yu, Z. Zheng, Z. Ding, X. Zhao, J. Yang, Y. Wei, Y. Chen, S. Yu, Ultra-low threshold continuous-wave quantum dot mini-BIC lasers. *Light Sci. Appl.* **12**, 100 (2023).

59. H. Jeon, J. Ding, A. V. Nurmikko, H. Luo, N. Samarth, J. Furdyna, Low threshold pulsed and continuous-wave laser action in optically pumped (Zn,Cd)Se/ZnSe multiple quantum well lasers in the blue-green. *Appl. Phys. Lett.* **59**, 1293–1295 (1991).

60. S. Ivanov, S. Sorokin, S. Gronin, I. Sedova, A. Vainilovich, E. Lutsenko, True green and yellow low-threshold II-VI laser heterostructures for II-VI/III-N laser diode converters, in *Conference on Lasers and Electro-Optics* (Optica Publishing Group, 2015).

61. T. J. S. Evans, A. Schlaus, Y. Fu, X. Zhong, T. L. Atallah, M. S. Spencer, L. E. Brus, S. Jin, X.-Y. Zhu, Continuous-wave lasing in cesium lead bromide perovskite nanowires. *Adv. Opt. Mater.* **6**, 1700982 (2018).

62. Q. Shang, M. Li, L. Zhao, D. Chen, S. Zhang, S. Chen, P. Gao, C. Shen, J. Xing, G. Xing, B. Shen, X. Liu, Q. Zhang, Role of the exciton-polariton in a continuous-wave optically pumped $CsPbBr_3$ perovskite laser. *Nano Lett.* **20**, 6636–6643 (2020).

63. P. Brenner, O. Bar-On, M. Jakoby, I. Allegro, B. S. Richards, U. W. Paetzold, I. A. Howard, J. Scheuer, U. Lemmer, Continuous wave amplified spontaneous emission in phase-stable lead halide perovskites. *Nat. Commun.* **10**, 988 (2019).

64. J.-H. Kim, M. Inoue, L. Zhao, T. Komino, S. Seo, J.-C. Ribierre, C. Adachi, Tunable and flexible solvent-free liquid organic distributed feedback lasers. *Appl. Phys. Lett.* **106**, 053302 (2015).

65. G. Tsiminis, Y. Wang, A. L. Kanibolotsky, A. R. Inigo, P. J. Skabara, I. D. W. Samuel, G. A. Turnbull, Nanoimprinted organic semiconductor laser pumped by a light-emitting diode. *Adv. Mater.* **25**, 2826–2830 (2013).

66. B. S. Karunathilaka, U. Balijapalli, C. A. M. Senevirathne, Y. Esaki, K. Goushi, T. Matsushima, A. S. D. Sandanayaka, C. Adachi, An organic laser dye having a small singlet-triplet energy gap makes the selection of a host material easier. *Adv. Funct. Mater.* **30**, 2001078 (2020).

67. B. K. Yap, R. Xia, M. Campoy-Quiles, P. N. Stavrinou, D. D. C. Bradley, Simultaneous optimization of charge-carrier mobility and optical gain in semiconducting polymer films. *Nat. Mater.* **7**, 376–380 (2008).

68. C. Czekalla, C. Sturm, R. Schmidt-Grund, B. Cao, M. Lorenz, M. Grundmann, Whispering gallery mode lasing in zinc oxide microwires. *Appl. Phys. Lett.* **92**, 241102 (2008).

69. M. H. Huang, S. Mao, H. Feick, H. Yan, Y. Wu, H. Kind, E. Weber, R. Russo, P. Yang, Room-temperature ultraviolet nanowire nanolasers. *Science* **292**, 1897–1899 (2001).

70. S. C. Su, H. Zhu, L. X. Zhang, M. He, L. Z. Zhao, S. F. Yu, J. N. Wang, F. C. C. Ling, Low-threshold lasing action in an asymmetric double ZnO/ZnMgO quantum well structure. *Appl. Phys. Lett.* **103**, 131104 (2013).
